# Machine learning based attribution mapping of climate related discussions on social media

**DOI:** 10.1038/s41598-022-22034-1

**Published:** 2022-11-08

**Authors:** Akshay Kaushal, Animesh Acharjee, Anandadeep Mandal

**Affiliations:** 1 HSBC Global Research, HSBC Global Banking and Markets, Bangalore, India; 2 Institute of Cancer and Genomic Sciences, University of Birmingham, Birmingham, B15 2TT UK; 3 Department of Finance, Birmingham Business School, University of Birmingham, Birmingham, B15 2TT UK

**Keywords:** Climate sciences, Climate change

## Abstract

A united front from all the stakeholders including public, administration and academia alike is required to counter the growing threat of climate change. The recent rise of social media as the new public address system, makes it an ideal source of information to assess public discussions and responses in real time. We mine c.1.7 m posts from 55 climate related subreddits on social media platform Reddit since its inception. Using USE, a state-of-the-art sentence encoder, and K-means clustering algorithm, we develop a machine learning based approach to identify, store, process and classify the posts automatically, and at a scale. In the broad and multifaceted theme of climate change, our approach narrows down the focus to 10 critical underlying themes comprising the public discussions on social media over time. Furthermore, we employ a full order partial correlation analysis to assess the relationship between the different identified themes. We show that in line with Paris Agreement, while the *climate science* community has been successful in influencing the discussions on both the causes and effects of climate change, the *public administration* has failed to appropriately communicate the causes of climate change and has been able to influence only the discussions on the effects of it. Hence, our study shows a clear gap in the public communication by the administration, wherein counter-intuitively less emphasis has been given on the drivers of climate change. This information can be particularly beneficial to policymakers and climate activists in decision making as they try to close the gap between public and academia.

## Introduction

Understanding climate change is crucial to building sustainable societies and paving the path to growth and stability for future generations. The discussions on social media^1^ form a valuable source of unstructured^2^ data that is growing at an unprecedented rate^3^. Many studies have been conducted using unstructured text data and Natural Language Processing (NLP)^4,5^ tools. The aim is mainly to gain additional insights on various facets of the social media interactions that would otherwise be impossible with structured^6^ data alone. Social media has increasingly become an integral part of modern society^7^, as it reflects the response and opinions of the public regarding any event or cause in real-time. The users can consume^8^ large amounts of content on a plethora of themes from a variety of different platforms and exercise their freedom of expression^9^ openly or even anonymously^10^ if needed. In addition, a large number of active users network, speed of engagement^11^, and ease with which information can be accessed and shared on social media make it a very powerful medium of communication in modern society, to the extent that it is even dubbed as “new public address system^12^” as it continues to be adopted by leaders around the world. Considering the explosion that has been seen in the adoption of social media^13^ in the last decade, the influence of leaders on climate change attitudes of the general public^14^ is only likely to increase with time. Hence, collecting and analysing this abundant source of information is becoming increasingly crucial from various stakeholders’ perspectives ranging from policymakers to climate activists and even the public at large.

While Twitter^15^ has been used majorly as a source in social media and climate change related studies due to its ease of access, location tagging and real time availability of data in large quantities, there are limitations when it comes to conducting studies over longer time spans (more than 30 days^16^). Moreover, there have been relatively few studies^17–20^ which focused on other sources for such information and time spans.

Social media in relation to climate change has been studied from a variety of perspectives in the past. While on the operational side, the real-time updates on social media have been used to develop disaster response systems^21^, on the social side, the written words on social media have helped in assessing whether any climate related topic increases or decreases the happiness of the users over a given period of time^4^. These discussions and expressed sentiments on social media not only serve as a proxy for assessing the psychological impact of climate related events on the general population but also help in assessing how far the concerns are lagging in terms of climate change or energy issues^22^. Sentiment analysis has also found its application in generating predictive flight recommendations based on online consumer reviews^23^, and it remains a significant part of the workflow in recent social media related applications across multiple sectors ranging from healthcare, crime, travel, finance, and academia^24^.

There have been visible links observed between social media, and the public awareness and engagement in relation to climate change, wherein climate change related events have been studied in conjunction with rising trends in people’s searches on the internet in connection to those events^25^. Moreover, public awareness plays an important role in the legislation as well as implementation of climate related policies^26^. Clear communication with a targeted and tailored information provision from both policymakers and public alike is required to reduce carbon dependency at the source^27^. As even though the policymakers are responsible for decision making, it is the public who has to follow the guidelines eventually. Thus, the sooner the policymakers or the climate change activists have an understanding of the level of engagement of the public with respect to various climate change events/policies, the better they can devise the strategies to draw out the best social response out of them. Topic modelling remains one such powerful text mining tool^28^, which enables the policymakers to track the level of public engagement with respect to any such topic in real-time^29–32^. However, the majority of the past studies focused on a single facet of climate change at a time^22,29,33^, such as natural disasters, carbon taxation, energy etc., and there remains a lack of comprehensiveness in terms of limited sources of information used, shorter time spans covered^17–20^, limited innovation in terms of methods used^34,35^ such as Latent Dirichlet Allocation, Non-negative matrix factorization etc. across studies as such. With this paper, we aim to provide an innovative and state-of-the-art machine learning based approach to build a comprehensive analytical view around the climate change related discussions on social media over a longer time span of c.14 years, narrowing down the focus to 10 critical areas within the umbrella theme of climate change.

By identifying the key areas of discussions and the proportion in which they are being discussed in real-time can aid the policymakers bridge the gap between policy development and the respective social implications and response. For example, despite being an effective emission reduction policy, carbon taxation^36^ remains unpopular among masses mainly due to lack of trust in government, education, and the perceptions of taxation’s impact^33^. However, by knowing the underlying themes of discussions, the administrative programs could be designed in such a way that addresses the key concerns and promotes the factors that get less traction from the general populace but are more important from policy and climate change perspective and vice-versa. For instance, if lack of trust in government is in question, public awareness campaigns could be specifically focused on increasing transparency on policies and investments side^37^. And if lack of education is the concern, efforts could be concentrated on educating the public about the benefits of the same^38^. Hence, our approach can be used as a lever to assess how far the concerns are lagging/leading from a selected benchmark with respect to any critical climate change theme, and subsequently the climate related programs could be designed and budgeted to achieve the most desirable social response. The key advantage of this social media based system is that the social response to any new topic of discussion can be recorded in real-time^39^ and the processes/designs can be adjusted on the fly as per requirement. Furthermore, it can help in assessing public sentiments with respect to various topics of discussion in real-time, which in turn can be used as an input in the recommendation systems^23,40^ for decision makers, who are responsible for devising policies and public campaigns.

**Figure 1:**
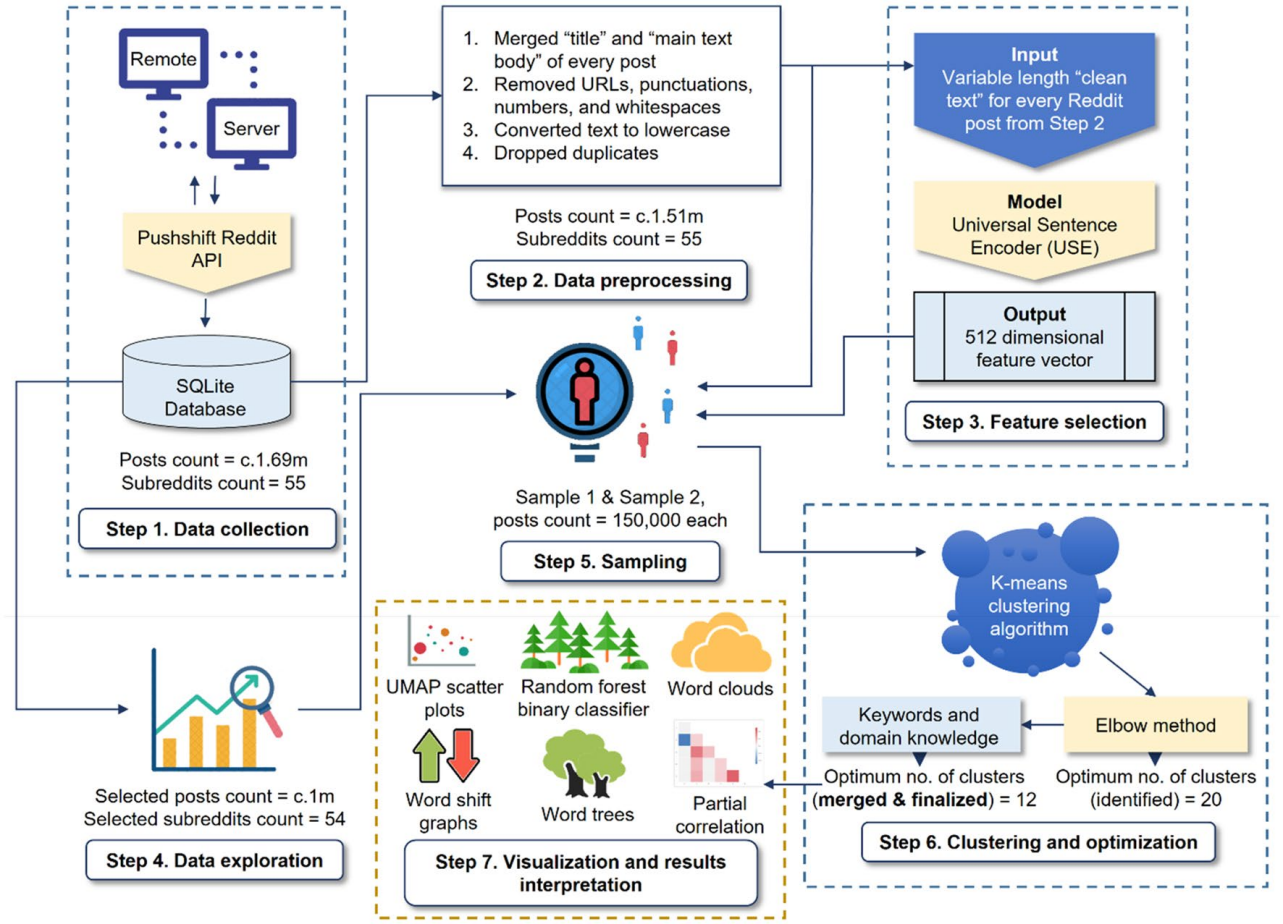
Workflow of the analytics platform and evaluation of the model from the data gathered. The figure provides a walkthrough of all the steps involved in this study beginning from *Data collection* (Step 1) to *Visualization and results interpretation* (Step 7). The solid arrows depict the direction of the flow of information between the different steps. The squares with dotted lines depict the boundary of a given step and the individual components within.

This paper aims to support the research in this area by providing a unique approach to generating insights from abundant social media text data. We collected c.1.7 million climate related text posts from Reddit^41^, an American social media platform for communities, across c.0.2 million users who posted in 55 different climate related communities (mainly known as *subreddits*) over the period between Jan 2008–Jun 2021. Using Universal Sentence Encoder (USE)^42^, a state-of-the-art model for encoding sentences into embedding vectors, we develop a machine learning^43^ pipeline to identify, process and classify the climate related posts on Reddit automatically, at a scale, and without any human intervention (Fig. 1). We employ K-means^44^, an unsupervised^45^ machine learning algorithm, to obtain distinct clusters within the collected posts over time. Further, we employ Random forest based binary classifier^46^, a supervised^47^ machine learning algorithm, to identify the underlying features of discussions within the identified clusters [climate related themes] over the period. In order to measure the strength of the relationship between these underlying themes over time, we perform a full order partial correlation analysis^48^ on every possible combination of candidate pair separately, while controlling for the effect of the rest of the themes. Finally, we use a variety of visualization techniques including dimensionality reduction by Uniform Manifold Approximation and Projection (UMAP)^49^, Word clouds^50^, interactive Word trees^51^ and Word shift graphs^52^ to generate snapshots of the output from various viewpoints.

The proposed pipeline provides us with valuable insights as to how the general population interacts and perceives information related to climate at large, which we believe could have major policy and social implications. While knowing the underlying themes around climate related discussions provide us with the list of areas that are in focus of the general public, the proportion in which they influence the discussions and the timing of it provides us with insights^53^ as to what causes shift in the climate related concerns of the general public, why it happens, when it happens, and to what extent it influences the discussions on social media. In doing so, we establish a new paradigm for assessing the climate related discussions on social media.

The paper is structured as follows: the data collection, exploration and details on methods employed in different steps of the workflow are described in the *Materials and Methods* section. The output of the analysis and its interpretation takes place in the *Results* section, followed by a final section on *Discussions and conclusion*.

## Materials and methods

### Data collection

We use Pushshift Reddit API^54^ to retrieve the climate related posts from Reddit. Pushshift is an open source collection and archiving platform for Reddit data that is queryable, provides larger single query limits as compared to Reddit API^55^, gets updated in real-time and contains historical data since Reddit’s inception. The database is freely accessible, reusable and interoperable due to its availability in widely accepted JSON^56^ format.

A total of 55 climate related key subreddits were identified on Reddit manually. We searched for word “climate” on Reddit and checked the results for top listed subreddits based on relevance. After a careful inspection of all the subreddits found in the extended search results, we finalised 55 subreddits which were closely associated with climate theme (Extended Table S1). All of the c.1.7 m posts made in these subreddits by c.0.2 m users since 2008 were downloaded using Pushshift API by looping through the list of subreddits using *before* and *after* timestamps, one subreddit at a time, and saved in an SQLite^57^ database for further processes.

The following six variables were captured for each text post; (1) timestamp, (2) title, (3) main text body, (4) author, (5) link to the original post on Reddit, and (6) subreddit in which the post appeared. We also assign unique IDs to every post captured so that the details pertaining to any single post can be identified and fetched from the SQLite database later with ease.

### Data preprocessing

Considering the structure of various posts, it was realized that most of the users type their main idea in the *title* of the post itself, with only occasional explanations/substantiations ranging from small one-liners to paragraphs in the *main body* of the post. Hence, for the sake of uniformity across the dataset, and to avoid any kind of data exclusions for cluster analysis, we merge the text from the *title* and *main body* of each post to form a *single text body*.

Since our focus is to identify the underlying themes of the discussions within the communities, we do further cleaning of the text to remove the extra information such as punctuation marks, URLs, numbers and whitespaces which is of least importance for our use case. Further, as the Reddit posts lack any formal writing standard, in order to maintain consistency and reduce sparsity of the data, we convert all of the text into lowercase and remove all the duplicates, which leaves us with c.1.5 m posts from 55 subreddits to be used as an input in the following step.

### Feature selection

In this step, we encode the *cleaned text* posts—obtained from the previous step—into feature vectors to be used in unsupervised machine learning^45^ model. While there are many methods^58^ available to generate feature vectors, we use state-of-the-art pre-trained Universal Sentence Encoder^42^ (USE) model which is publicly available in Tensorflow-hub^59^ for two reasons: (1) it works on character level and can handle polysemy. Thus, it can handle unseen words, and vector output obtained from the model will encapsulate the meaning of every word/sentence it sees. (2) It is designed to be a general-purpose model and provides a single fixed 512-dimension vector output for every text input provided ranging from a single word to a list of sentences, which is apt for our dataset with high variation in text input lengths.

Due to memory constraints on a machine with 16 GB RAM, instead of committing all of c.1.5 m *cleaned text* posts to memory at once, we subdivide the whole corpus into groups of 1000 posts each and obtain the corresponding USE feature vectors for each group one at a time. We save the IDs of the posts and corresponding feature vectors on the local disk for further processing.

### Data exploration

**Figure 2:**
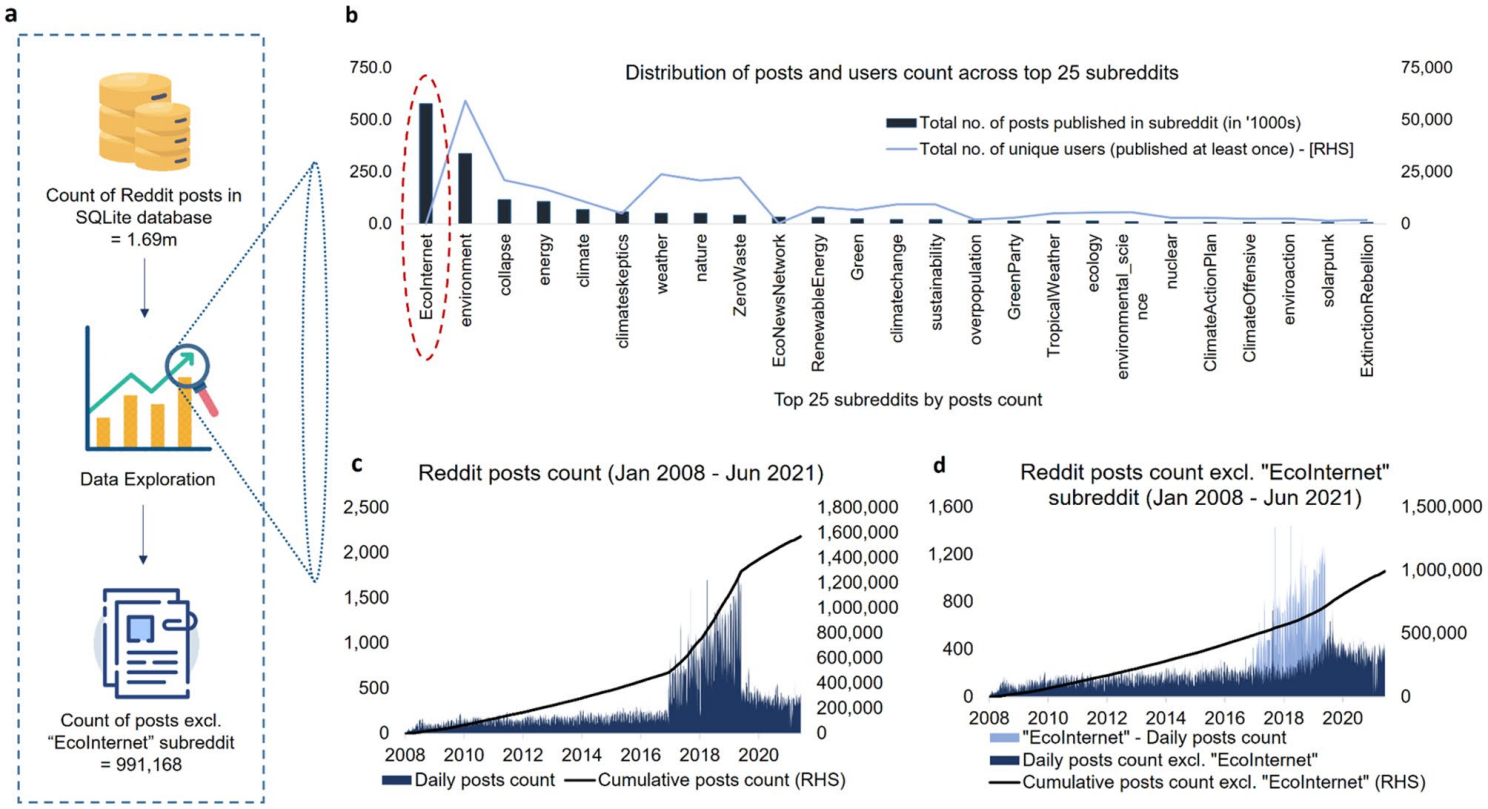
Data exploration and finalization. Overall we observe a gradual increase in the posts count over the study period except for a single time period from Jan 2017 to Jun 2019 (**c**). From the total number of c.1.7 m posts collected from 55 climate related subreddits, the *EcoInternet* subreddit alone accounted for c.35% of the posts (**b**). However, it was active only for a relatively short period of time from Jan 2017 to Jun 2019 (**d**), and only a single Reddit user namely *EcoInternetNewsfeed* was responsible for publishing c.99% of the total posts within *EcoInternet* during that time frame, resulting in a very small and disproportionate user base as compared to other subreddits—highlighted using red dotted line ellipse above (**b**). After excluding the data from *EcoInternet* subreddit, we observe a gradual increase in posts count over the entire study period (2008–2021) (**d**). Considering it as an outlier, we exclude all of the posts from *EcoInternet* subreddit, and finalize 991,168 posts (**a**) from the rest of the 54 subreddits for further processing.

Before moving on to modelling and optimization exercise, we also employ preliminary exploration of the raw data saved in the SQLite database (Fig. 2a) to assess the structure and general distribution of the posts, and retrieve useful information to be used as an input in the following steps of the workflow. From the distribution of posts and users count across the subreddits (Fig. 2b), we observe that out of the total 55 climate related subreddits identified manually, most of the users traffic is concentrated in around top 25 subreddits (by posts count) only, wherein top 10 subreddits account for c.86% of the total posts published and top 25 subreddits account for c.98% of the total posts published with the rest of the 30 subreddits accounting for merely c.2% of the total posts published from Jan 2008 to Jun 2021. However, by taking a closer look at the distribution, we realize that except for *EcoInternet* subreddit (*highlighted with red dotted line ellipse in Fig. **2*b), the posts and users count is fairly distributed across the rest of the 54 subreddits, wherein the number of posts has a very high correlation of 0.93 with the number of users such that the number of posts rise with number of users and vice-versa, which is within expectations as with high number of users the discussions and thereby posts count is expected to rise as well.

Although, in case of *EcoInternet* we observe that even though the subreddit ranks on top in terms of total posts count and account for c.35% of the total posts collected, the relative number of users publishing in the subreddit is unusually low (only 14 number of users), and even among those users, only a single username *EcoInternetNewsfeed* was responsible for publishing c.99% of the total posts within the subreddit. Moreover, by observing the daily count distribution of the posts with and without *EcoInternet* (Fig. 2c,d), we realize that overall, there is a gradual increase in the posts over the entire study period except for a single time period from Jan 2017 to Jun 2019, when we see an unusually high activity (Fig. 2c). A closer look at the underlying data reveals that *EcoInternet* subreddit, specifically a single user from the subreddit alone, is responsible for that unusually high activity during that relatively short time-period (Fig. 2d). After separating *EcoInternet* from the rest of the subreddits, we observe a gradual increase in daily posts count over the entire study period. Hence, taking all of this into account, in addition to the consideration that a subreddit which contains posts mostly from a single user alone cannot be considered a community of users, we treat *EcoInternet* as an outlier, and thus exclude all its posts going forward. This exercise leaves us with a total of 991,168 posts from 54 subreddits to be used as an input in further processes.

### Application of the unsupervised and supervised algorithms

We use K-means^44^, an unsupervised machine learning algorithm, to identify distinct clusters within our dataset, with the main advantage of this approach being a large constant-factor speedup that is gained by training with K-means and easy implementation at large scale^60^. One of the limitations however is that K-means, similar to other clustering algorithms, takes in all of the input at once. But considering the huge size of the corpus (c.10 GB) following the USE features selection and data exploration, it was not possible to commit all of the dataset to memory at once as memory requirement for running machine learning pipeline exceeds significantly afterwards. To overcome this limitation, time-dependent sampling was employed, wherein the whole dataset from 2008 to 2021 was subdivided into monthly subgroups, and a random 30% of the posts were selected from each month, thereby creating two distinct non-overlapping sample time-series of size 0.15 m each (with c.15% posts in each sample). It was done based on the underlying assumption that similar discussions should have happened during the same time period within the subreddits and among different users.

Once sampling is done, the next step is to run the K-means clustering algorithm on both samples. We use the Scikit-learn machine learning library^61^ for python to achieve this objective, wherein the feature vectors for each sample are provided as input for training the model and the corresponding cluster labels are obtained as output. However, one of the biggest challenges in running K-means clustering algorithm is to decide on the value of *k* i.e. the number of optimum clusters within the dataset. We employ a two-step approach to tackle this problem; (1) optimize the number of clusters using the Elbow method^62^, (2) manually optimize the clusters obtained from the previous step further based on similarity of the underlying keywords (mainly unigrams and bigrams) obtained within the clusters and domain knowledge.

**Figure 3:**
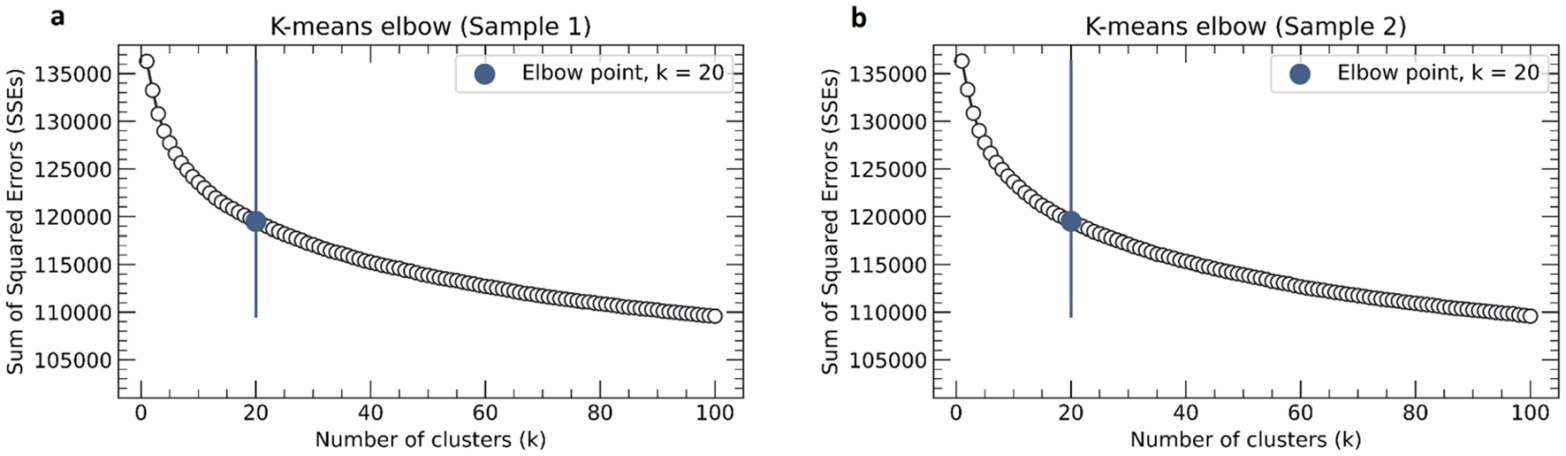
Optimizing the number of clusters (k) using the Elbow method. It is a heuristic technique used to determine the number of optimum clusters in a given sample. Here, the explained variation (SSE) has been plotted as a function of the number of clusters for both Sample 1 (**a**) and Sample 2 (**b**) obtained earlier. The blue vertical lines at k = 20 denote the elbows of the plotted lines, signifying 20 numbers of optimum clusters in both Sample 1 and 2, beyond which SSE starts to decrease in a somewhat linear fashion as seen in both left as well as right plot.

The key objective of the K-means algorithm on each iteration is to minimize the Sum of Squared Error (SSE). Hence, to obtain the optimum *k*, we run the K-means clustering algorithm for a number of values of *k*, ranging from 1 to 100 for each sample and plot the respective SSE versus k (Fig. 3). We obtain almost similar outcomes in case of both samples 1 and 2 with the Elbow somewhere around *k* = 20, after which SSE starts to decrease in a somewhat linear fashion.

Once we have the optimum number of *k* as 20 from Elbow method, we further optimize the clusters by having a closer look at the list of keywords within every cluster to identify the underlying themes driving those cluster differences as well as similarities if any thematically or otherwise. After a close inspection of the underlying keywords, we merge and narrow down the optimum number of clusters further to 12 from 20 earlier, and thereby finalize 12 different underlying themes comprising the climate related discussions on the social media platform Reddit.

In order to determine important features and their respective weights for every optimized cluster in Sample 1 and 2, we train a Random forest based binary classifier for every cluster separately. In every iteration, the cluster for which important features were to be determined was labelled as 1, while labelling the rest of the clusters in the sample as 0. It allowed us to identify the key words/themes and their respective weights which were driving the discussions within all of the optimized clusters separately.

### Application of the full order partial correlation analysis

For the purpose of measuring the strength of the relationship between different identified optimized clusters [themes], we perform a full order partial correlation analysis^48^ on the optimized clusters time series obtained from the previous step. One of the major benefits of using this approach is that it enables us to differentiate between direct and indirect interactions of the underlying climate related themes^63^, thereby allowing us to focus on clear one-to-one underlying interconnections between variables which is not possible in the case of zero order correlation. Although, this approach suffers from Berkson’s paradox^64^, and there remains a possibility of false observations of negative correlation between different cluster pairs^65^. We employ it in our workflow mainly because it allows us to observe the collective behaviour of multiple climate related themes in one form, and it is relatively simpler in terms of its application.

We create a list of all the possible combinations of selected optimum cluster pairs (n = 10, number of combinations; ^n^C_2_ = ^10^C_2_ = 45) and iterate over it one at a time, while controlling for the effect of the rest of the clusters. We obtain a full order partial correlation matrix and a *p* value matrix as the output (Fig. 15).

### Data visualization

**Figure 4:**
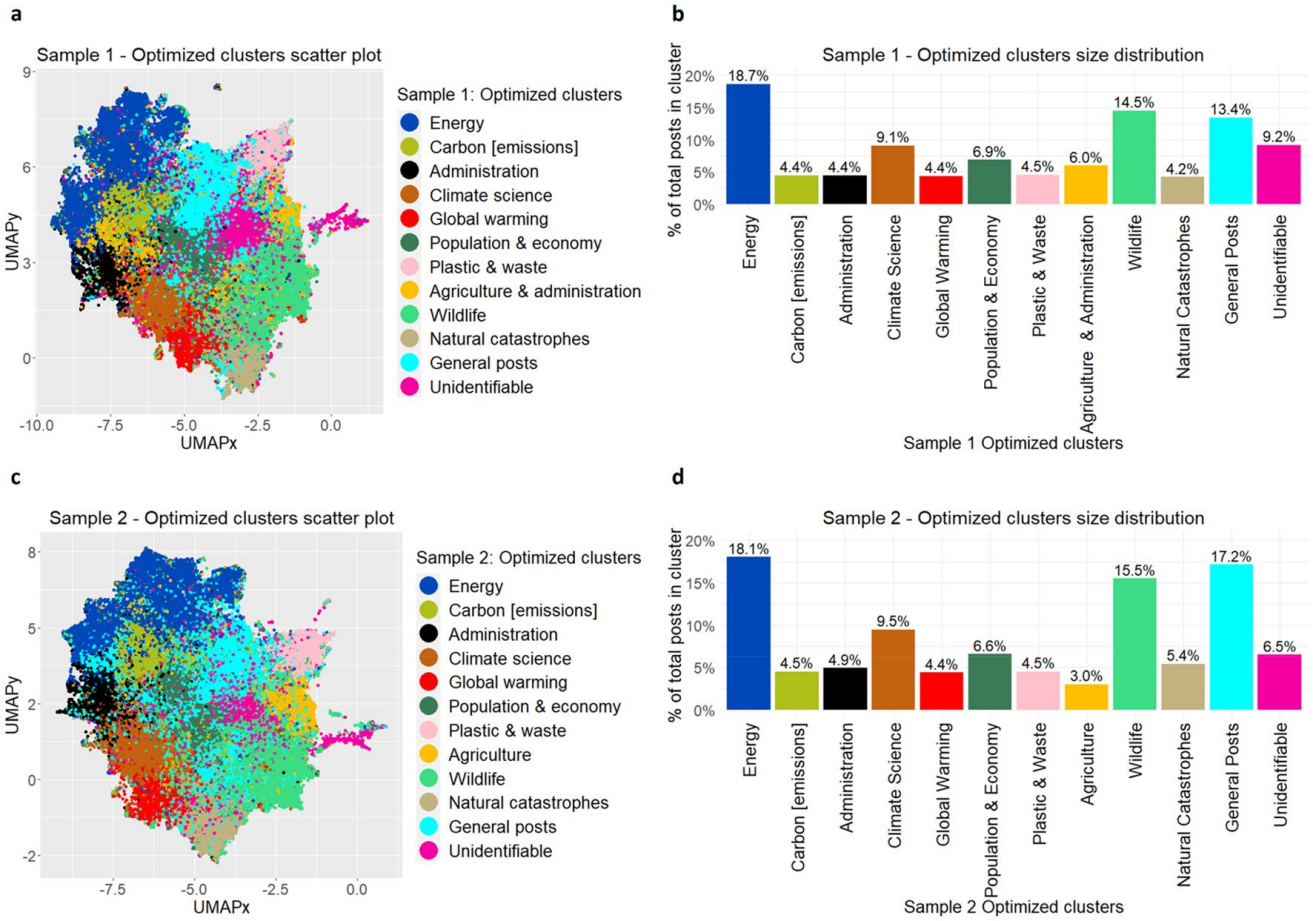
Final optimized clusters. Shown here are the final optimized clusters obtained for both Sample 1 (**a**, **b**) and Sample 2 (**c**, **d**). On left hand side are the scatter plots visualized using UMAP model, by projecting the 512D USE feature vectors on a 2D space using two UMAP components denoted as UMAPx and UMAPy representing the x-axis and y-axis of the scatter plots, with different colours representing the unique optimized clusters for Sample 1 (**a**) and Sample 2 (**c**). On the right-hand side are the size distribution column charts for the same optimized clusters with corresponding colours showing the percentage of total number of Reddit posts contained within those clusters obtained from Sample 1 (**b**) and Sample 2 (**d**).

As the USE feature vectors have 512 dimensions for each text post, it is not possible to visualize it straightaway in a 2D space. Hence, we use the Uniform Manifold Approximation and Projection (UMAP)^49^ model to achieve this objective. One of the key advantages of UMAP is that it allows to train a model on a sample dataset and once it has optimized the embedded space, it can be used to transform the rest of the data into the learned space and is therefore reusable in production. We provide 512D USE vectors as input and obtain two components, (titled UMAPx and UMAPy for the sake of simplicity) as output from UMAP, thus allowing us to visualize the 12 optimized clusters in a 2D space (Fig. 4a,c).

Furthermore, in order to visualize the underlying text data, we generate word clouds, word shift graphs and word trees for all of the 12 optimized clusters in both the samples 1 and 2. These visualizations allow us to have an in-depth look at both the underlying similarities between each sample and the underlying differences between clusters within each sample.

### Data and scripts availability

The raw data is c.500 MB in size and is available from authors on request. The code used to produce the results is made available in a public repository^66^—https://github.com/akshaydnicator/ClimateChangeReddit.

## Results

### Identified the optimum number of clusters

We begin with the K-means elbow clustering method first, in our two-step clusters optimization approach. From the plot of SSEs versus Number of clusters (k) (Fig. 3), we observe that SSE starts to decrease in a somewhat linear fashion after k = 20 for both the samples 1 and 2. Moreover, both the samples show an almost similar trend line beginning with a steep fall and then gradually declining at a reducing rate. Hence, we denote the point on the vertical blue line as the elbow of the plot and consider 20 as the number of clusters for further optimization.

We do further inspection of the underlying keywords (mainly unigrams and bigrams) belonging to all the 20 clusters obtained earlier. While the order of the clusters differ between sample 1 and 2, we observed that both ended up with almost similar clusters with underlying themes and respective sizes except for a small number of differences. Thus, based on the similarities and differences identified between all 20 clusters found in sample 1 and 2, we merge several clusters, mainly starting with the clusters representing the *Energy* theme (Extended Data Fig. S3) such as *Electric Vehicles (EVs)/Alternative energy resources, Solar energy, Renewable energy excl. Solar, Nuclear energy and Oil & gas.* We also merge clusters representing the *Wildlife* theme such as those representing *Marine life, Animals/Forests and Nature in general.* Finally, we combine the posts from clusters related to *Sustainability, Socialization, General research and Mixed* themes together under the umbrella title *General posts.* Hence, finally we reduce our number of clusters to 12 from 20 for both the samples 1 and 2.

After the two steps of optimization of the number of clusters, we obtain the same number of clusters as 12 with almost identical underlying themes in case of both sample 1 and 2. The only major difference that stands out between the two is that while in Sample 1 we obtain a cluster with *Administration* as underlying theme and also another cluster with *Agriculture and administration* as the underlying theme i.e. a combination of both, in Sample 2 we obtain two clear distinct clusters with *Administration* and *Agriculture* as separate underlying themes. The same can be observed from the scatter plot (Fig. 4a), wherein a part of the yellow coloured *Agriculture and administration* cluster is tending to merge with the black coloured *Administration* cluster, as opposed to sample 2 (Fig. 4c), wherein we get distinct clusters for both *Administration* and *Agriculture*. However, we note that *Agriculture* remains as one of the prominent themes of interest for the general public and features quite consistently in the discussions on Reddit communities.

Overall, we identify 10 distinct climate related clusters/themes that get discussed extensively on Reddit in varying proportion (Fig. 4b,d), dominated mainly by *Energy (c.18%)*, followed by *Wildlife (c.15%), Climate science (c.9%), Population & economy (c.7%), Administration (c.5%), Natural catastrophes (c.5%), Carbon [emissions] (c.5%), Agriculture (c.5%), Global warming (c.4%), and Plastic & waste (c.4%)*. In addition, two more clusters are identified viz. *General posts (c.15%)*—contains climate related posts that do not touch any single theme specifically and discuss climate in general from a combination of perspectives, and *Unidentifiable (c.8%)*—contains posts that do not discuss climate in general and as such do not belong to any of the identified climate related clusters/themes. Further, within *Energy* cluster, the discussions are largely dominated by *Renewable energy (c.46%, o/w Solar energy*—*c.16% and others—c.30%)*, followed by *Oil & gas (23%)*, *Electric vehicles & alternative fuels (c.18%)*, and *Nuclear energy (c.13%)*. We also observe their individual monthly proportion time-series over time to see if there were any unusual activities among Reddit users over time due to certain specific events which are worth highlighting (Extended Data Fig. S3).

### Identified the underlying features of optimized clusters

**Figure 5:**
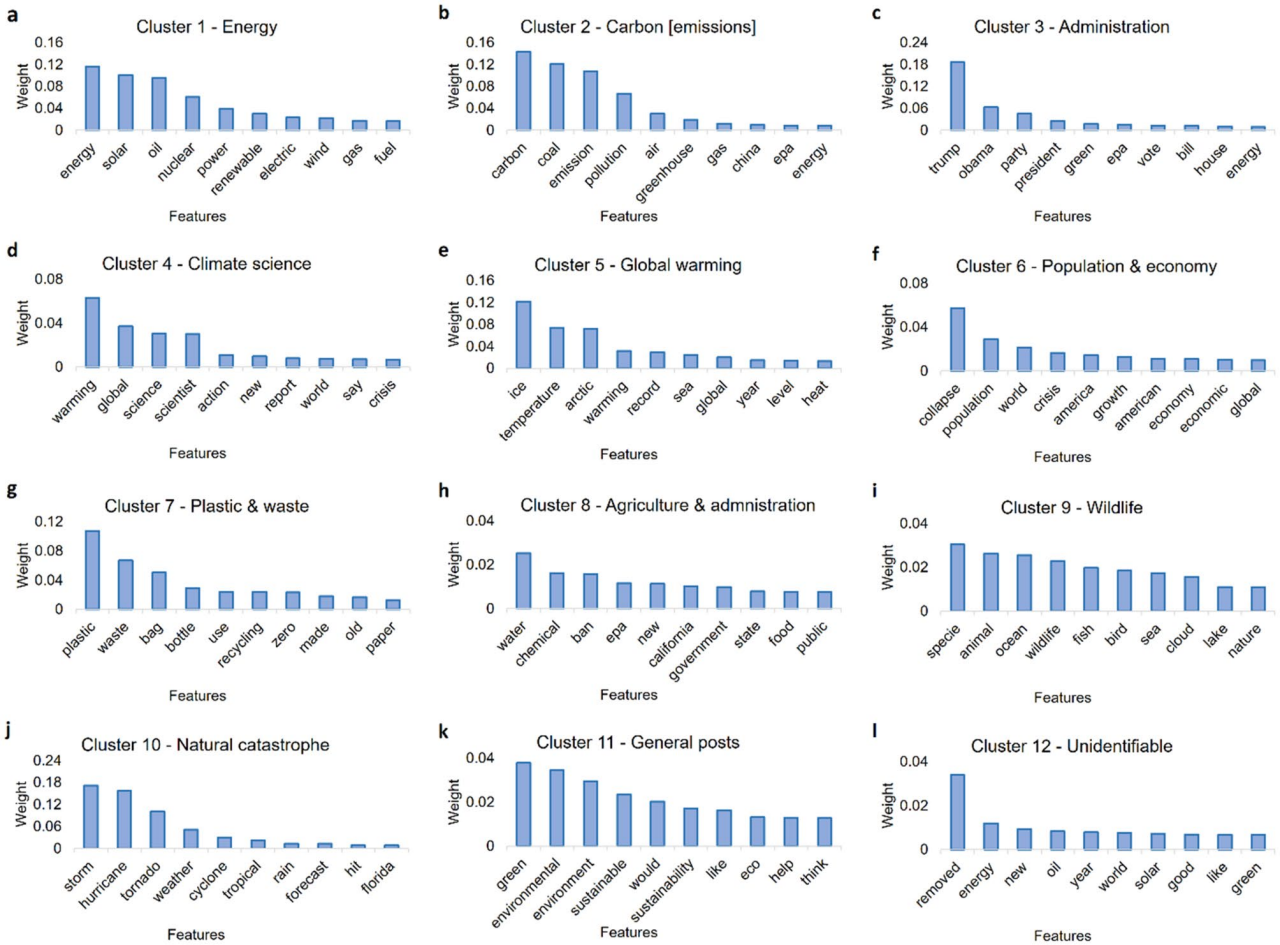
Showing the underlying features driving the discussions within the optimized clusters obtained for Sample 1. The underlying features are obtained by training a supervised learning-based Random forest binary classifier. From the output, we observe that all the clusters are composed of discussions related to unique themes and sub-themes within them. For instance, we obtain features such as solar, oil, nuclear, etc. in Energy cluster (**a**), and carbon, coal, emission in Carbon [emissions] cluster (**b**), whereas features such as trump, Obama, party, etc. dominate the Administration cluster (**c**). Similarly, warming, global, science, etc. comprise the Climate science cluster (**d**) whereas temperature, ice, arctic, etc. form the Global warming cluster (**e**). In the Population & economy cluster, features such as collapse, population, crisis, etc. (**f**) get the highest weightage, however, plastic, waste, bag, etc. get the highest weightage in the Plastic & waste cluster (**g**). In the Agriculture & administration cluster (**h**) features such as water, chemical, ban, etc. dominate the discussions, while in the Wildlife cluster (**i**), features such as specie, animal, fish, etc. dominate the discussions. Finally, storms, hurricanes, etc. dominate the Natural catastrophe cluster (**j**), whereas general climate-related terms such as green, environmental, etc. form the General posts cluster (**k**). The Unidentifiable cluster (**l**) on the other hand does not have a higher weightage assigned to any climate-related theme in general.

**Figure 6:**
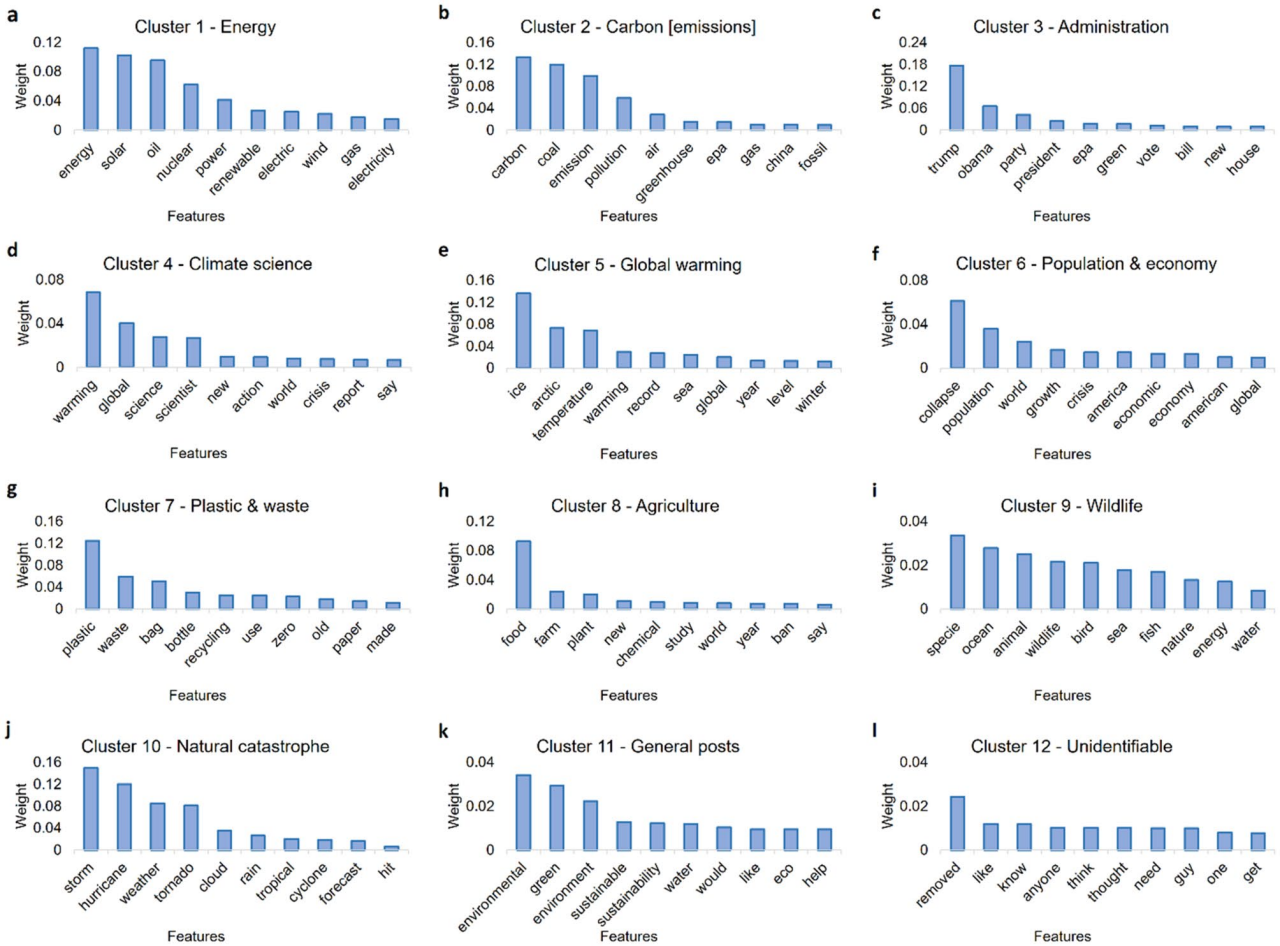
Showing the underlying features driving the discussions within the optimized clusters obtained for Sample 2. The underlying features are obtained by training a supervised learning-based Random forest binary classifier. From the output, we observe that all the clusters are composed of discussions related to unique themes and sub-themes within them. For instance, we obtain features such as solar, oil, nuclear, etc. in Energy cluster (**a**), and carbon, coal, emission in Carbon [emissions] cluster (**b**), whereas features such as trump, Obama, party, etc. dominate the Administration cluster (**c**). Similarly, warming, global, science, etc. comprise the Climate science cluster (**d**) whereas temperature, ice, arctic, etc. form the Global warming cluster (**e**). In the Population & economy cluster (**f**), features such as collapse, population, crisis, etc. get the highest weightage, however, plastic, waste, bag, etc. get the highest weightage in the Plastic & waste cluster (**g**). In the Agriculture cluster (**h**) features such as food, chemical, farm, etc. dominate the discussions, while in the Wildlife cluster (**i**), features such as specie, animals, fish, etc. dominate the discussions. Finally, storms, hurricanes,s, etc. dominate the Natural catastrophe cluster (**j**), whereas general climate-related terms such as green, environmental, etc. form the General posts cluster (**k**). The Unidentifiable cluster (**l**) on the other hand does not have a higher weightage assigned to any climate-related theme in general.

We train a Random forest based binary classifier for all of the 12 identified optimized clusters separately for both Sample 1 and 2 and obtain the important underlying features and their respective weights within those clusters. We observe that we get similar output in case of both the samples with similar features and weights dominating the discussions within the corresponding clusters in each sample (Figs. 5, 6). For instance, in case of *Energy* (Figs. 5a, 6a)*, Carbon [emissions]* (Figs. 5b, 6b)*, Administration* (Figs. 5c, 6c)*, Climate science* (Figs. 5d, 6d)*, Global warming* (Figs. 5e, 6e)*, Population & economy* (Figs. 5f, 6f), *Plastic & waste* (Figs. 5g, 6g), and *Wildlife* (Figs. 5i, 6i) clusters, we get exactly same first three features with almost similar weights driving the discussions within those clusters. In case of *Natural catastrophe* cluster, we observe slight shuffling of the features (Figs. 5j, 6j), but overall they stay the same for Sample 1 and 2. However, in case of *Agriculture [and Administration (for Sample 1)],* we see a clear difference in terms of underlying features of discussions within the two samples as features such as *water, ban, government *etc*.* (Fig. 5h) dominate the discussions in sample 1, whereas features such as *food, farm, plant *etc*.* (Fig. 6h) get the highest weightage in sample 2. Apart from that, as expected we observe general topics such as *environment, sustainability, eco *etc*.* (Figs. 5k, 6k) feature in the *General posts* cluster, whereas the *Unidentified* cluster comprises of random features unrelated to the climate change in general (Figs. 5l, 6l).

### Visualization and interpretation

**Figure 7:**
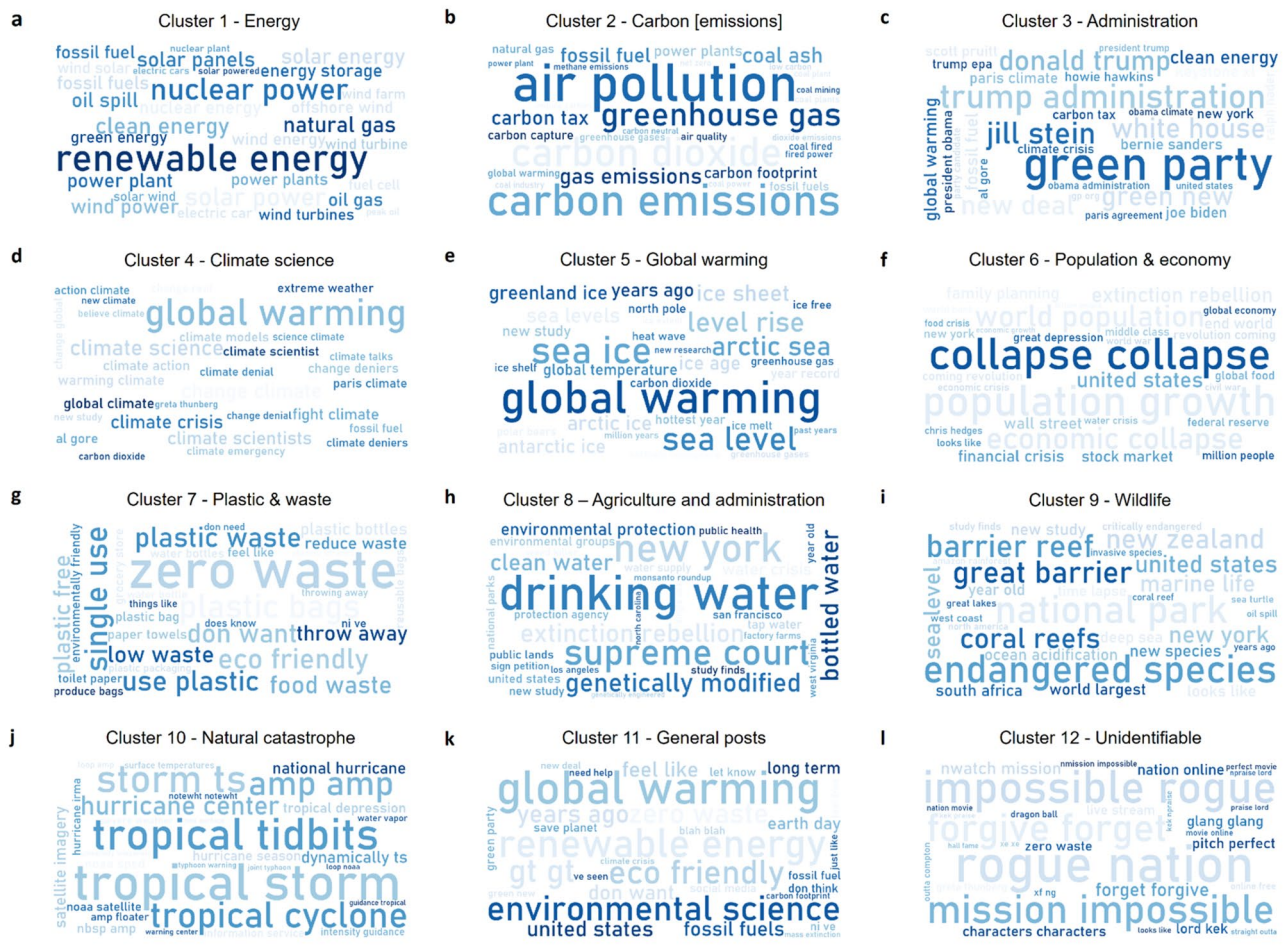
Sample 1 final optimized clusters in word clouds^50^. The word cloud for a given optimized cluster has been generated from top 30 bigrams^68^ featured in that cluster sorted by Tfidf-Vectorizer^69^ weights. It provides a bird's view into the underlying keywords belonging to all 12 clusters (**a**–**l**), specifically showing how distinguished the optimized clusters are in terms of their composition. For instance, “global warming” features in clusters 4 & 5 both (**d**, **e**), however by observing the other bigrams within the respective word clouds, we see that words like “climate science/scientists”, “climate denial” etc. dominate the Climate science cluster (**d**) while “sea ice”, “level rise” etc. - mainly the impacts of global warming - dominate the Global warming cluster (**e**). Moreover, we observe a clear distinct group of bigrams belonging to each optimized cluster thereby expressing the underlying theme distinctly except for General posts (**k**) and Unidentifiable (**l**) clusters. While the General posts cluster (**k**) contains mainly Reddit posts belonging to a wide range of themes of climate as opposed to discussing a specific theme as observed in other identified clusters, the Unidentifiable cluster (**l**) mostly contains posts which are having discussions of other than climate and thus do not belong to any specific climate theme or cluster.

**Figure 8:**
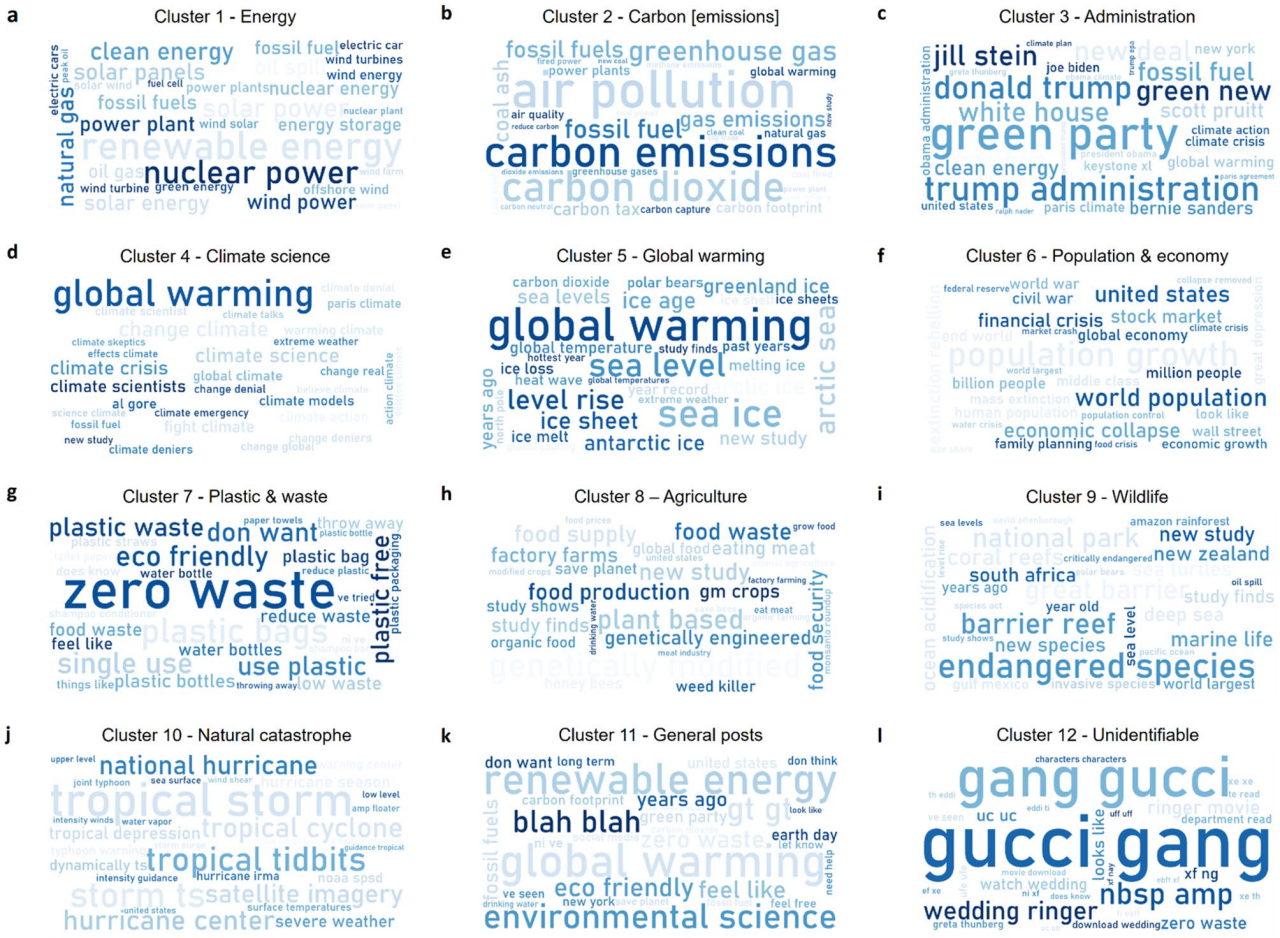
Sample 2 final optimized clusters in word clouds^50^. The word cloud for a given optimized cluster has been generated from top 30 bigrams^68^ featured in that cluster sorted by Tfidf-Vectorizer^69^ weights. It provides a bird's view into the underlying keywords belonging to all 12 clusters (**a**–**l**), specifically showing how distinguished the optimized clusters are in terms of their composition and how similar they are when compared with Sample 1 (Fig. 7). We observe a distinct group of bigrams belonging to each optimized cluster in Sample 2 similarly as observed in Sample 1 thereby expressing the underlying theme distinctly for each cluster. The only difference between Sample 1 & 2 was observed in the Agriculture cluster (**h**), whereby we observe that while in Sample 2, bigrams are able to express the agriculture theme clearly, in Sample 1 (Fig. 7h), we observe a mix of bigrams from agriculture and administration themes, and so the agriculture-related posts also contain text related to administration. The same can also be observed from scatter plots (Fig 4a, c), where in Sample 1, a part of the Agriculture cluster highlighted in yellow is mixed up with the Administration cluster highlighted in black as opposed to Sample 2, where both of the clusters are clearly distinct.

We further visualize the 12 optimized clusters identified for sample 1 and 2 using word clouds mainly comprised of top 30 bigrams (Figs. 7, 8) and top 30 keywords including both unigrams and bigrams (Extended Data Figs. S1, S2) to have a broader look at the underlying keywords and thereby themes that are being expressed by every cluster. We observe that overall, every keyword in the respective word clouds is able to clearly express the broader theme of the cluster and shows why it belongs to a specific cluster. Except that here as well, as observed previously, words such as “supreme court”, “water”, “genetically modified” etc. (Fig. 7h, Extended Data Fig. S1h) are part of the *Agriculture and administration* cluster in sample 1, as opposed to sample 2, wherein, words such as “food production”, “monsanto”^67^, “genetically engineered” etc. (Fig. 8h, Extended Data Fig. S2h) are part of the *Agriculture* cluster, thereby clearly expressing the overall theme. Also as expected, the *General posts* cluster contains keywords such as “world”, “global warming”, “environmental science” etc. (Figs. 7k, 8k, Extended Data Figs. S1k, S2k), thereby showing how the discussions within those clusters belong to a range of climate related themes instead of specific ones as observed in the rest of the 10 clusters. Moreover, keywords such “impossible rogue”, “watch”, “online” etc. in sample 1 (Fig. 7l, Extended Data Fig. S1l) and “gang gucci”, “wedding ringer” etc. in sample 2 (Fig. 8l, Extended Data Fig. S2l) show how the discussions within the cluster titled as *Unidentifiable* do not relate to any climate related theme and in fact do not discuss *climate* at all.

Apart from that, we observe words such as “renewable energy”, “nuclear”, “solar” etc. (Figs. 7a, 8a, Extended Data Figs. S1a, S2a) dominate the discussions related to *Energy* on Reddit, while “carbon emissions”, “coal”, “air pollution” etc. (Figs. 7b, 8b, Extended Data Figs. S1b, S2b) dominate the discussion related to *Carbon [emissions]*. In case of *Administration*, words such as “donald trump”, “green party”, “obama” etc. (Figs. 7c, 8c, Extended Data Figs. S1c, S2c) feature the discussions on Reddit climate communities. Notably, in the *Climate science* related discussions “global warming” features at the top of the discussions (Figs. 7d, 8d, Extended Data Figs. S1d, S2d), however words such as “climate science”, “climate crisis” etc. also feature in the discussions thereby showing how this cluster differs from the next cluster i.e. *Global warming,* which as expected features the “global warming” keyword at the top (Figs. 7e, 8e, Extended Data Figs. S1e, S2e), however also features keywords such as “sea ice”, “sea level”, “arctic” etc. to show that this cluster is mainly related to discussions on the impacts of the global warming, and hence discusses the theme from a different perspective as compared to *Climate science* cluster. Further, we observe that keywords such as “population growth”, “economic collapse”, “crisis” etc. (Figs. 7f, 8f, Extended Data Figs. S1f, S2f) dominate *Population & economy* cluster showing that mainly the discussions here relate to the concerns with respect to the rising global population and its related impacts on the climate and global economy at large. In the *Plastic & waste* cluster, words such as “plastic”, “zero waste”, “single use” etc. (Figs. 7g, 8g, Extended Data Figs. S1g, S2g) feature at the top, whereas in the *Wildlife* we observe words such as “endangered species”, “wildlife”, “national part” etc. (Figs. 7i, 8i, Extended Data Figs. S1i, S2i). Finally, in the case of the Natural *catastrophe* cluster we see that the discussions are mostly dominated by words such as “tropical storm”, “hurricane”, “weather” etc. (Figs. 7j, 8j, Extended Data Figs. S1j, S2j).

Thus, word clouds provide us with an overall snapshot of the optimized clusters obtained from Sample 1 and 2 over the entire study period.

### Optimum clusters time-series analysis

**Figure 9:**
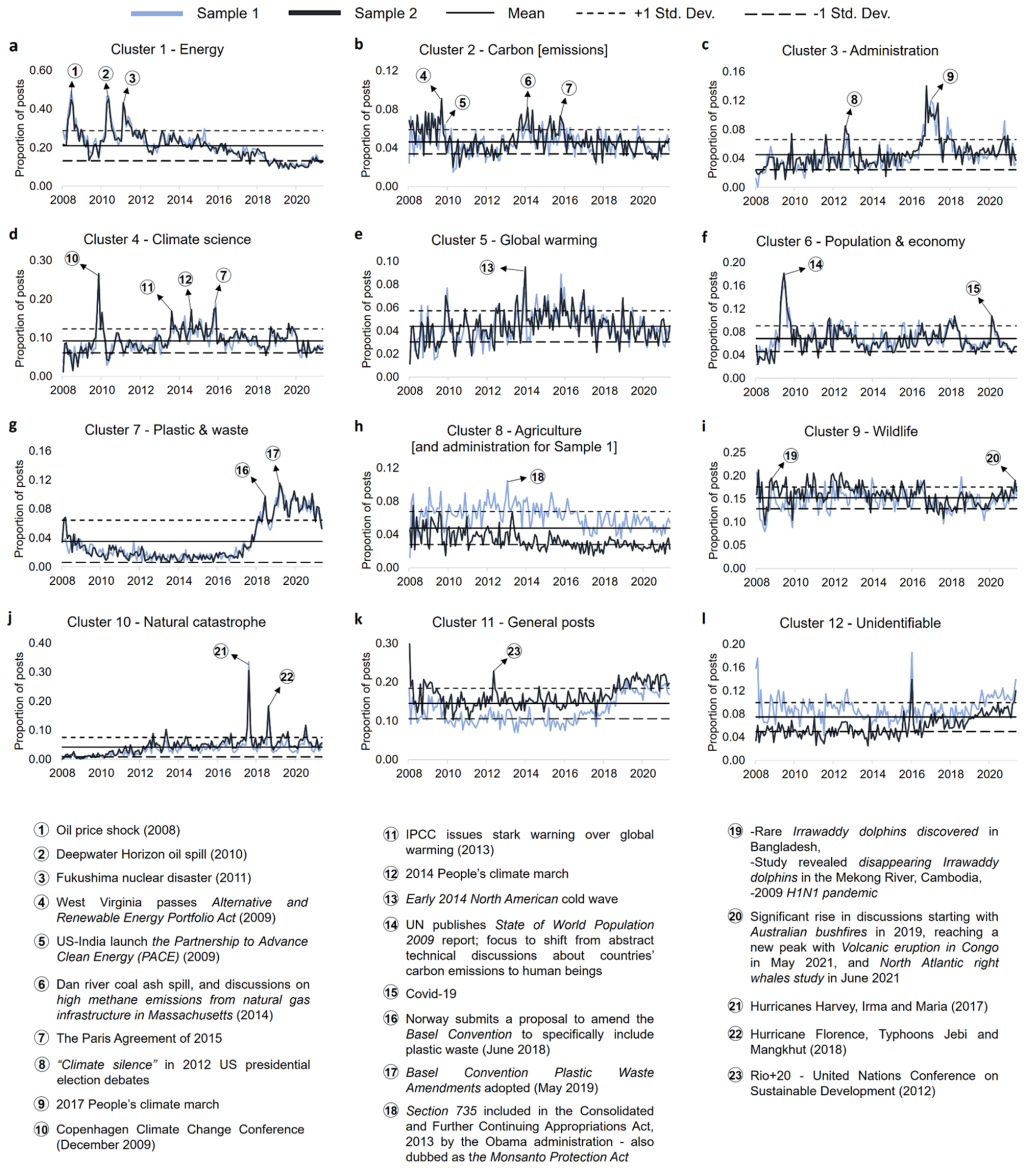
Proportion of posts in optimized clusters on a monthly basis (Jan 2008–Jun 2021). The figure shows the distribution of the proportion of the 12 optimized clusters on a monthly basis within sample 1 and 2 over the entire study period (**a**–**l**). The solid horizontal line depicts the overall mean of sample 1 and 2 whereas the two dotted lines above and below depict the ±1 sample standard deviation for both samples over the entire study period. All in all, within expectations, except for the three clusters namely Agriculture [and administration] (only Agriculture for sample 1) (**h**), General posts (**k**), and Unidentifiable (**l**), we observe that sample 1 and 2 time-series exhibit almost similar trend for the rest of the clusters (**a**–**g**, **i**, **j**), even in case of these three time-series, we observe a similar trend between sample 1 and 2 over time. This further strengthens our view that the underlying themes, on which the Reddit users are focussed in general, are uniform across the entire study period.

Moving forward, we plot the monthly proportion time-series of the optimized clusters (Fig. 9) to observe if there are any significant movements in users’ activity over time and visualize the changes in the underlying discussions using word shift graphs to identify the reasons for the movements in the same. To employ the word shift graphs, we compare the text posts from the periods with unusually high activity with the ones with normal or relatively low activity to observe the exact words responsible for the sudden shift in the quantum of user discussions during those times.

**Figure 10:**
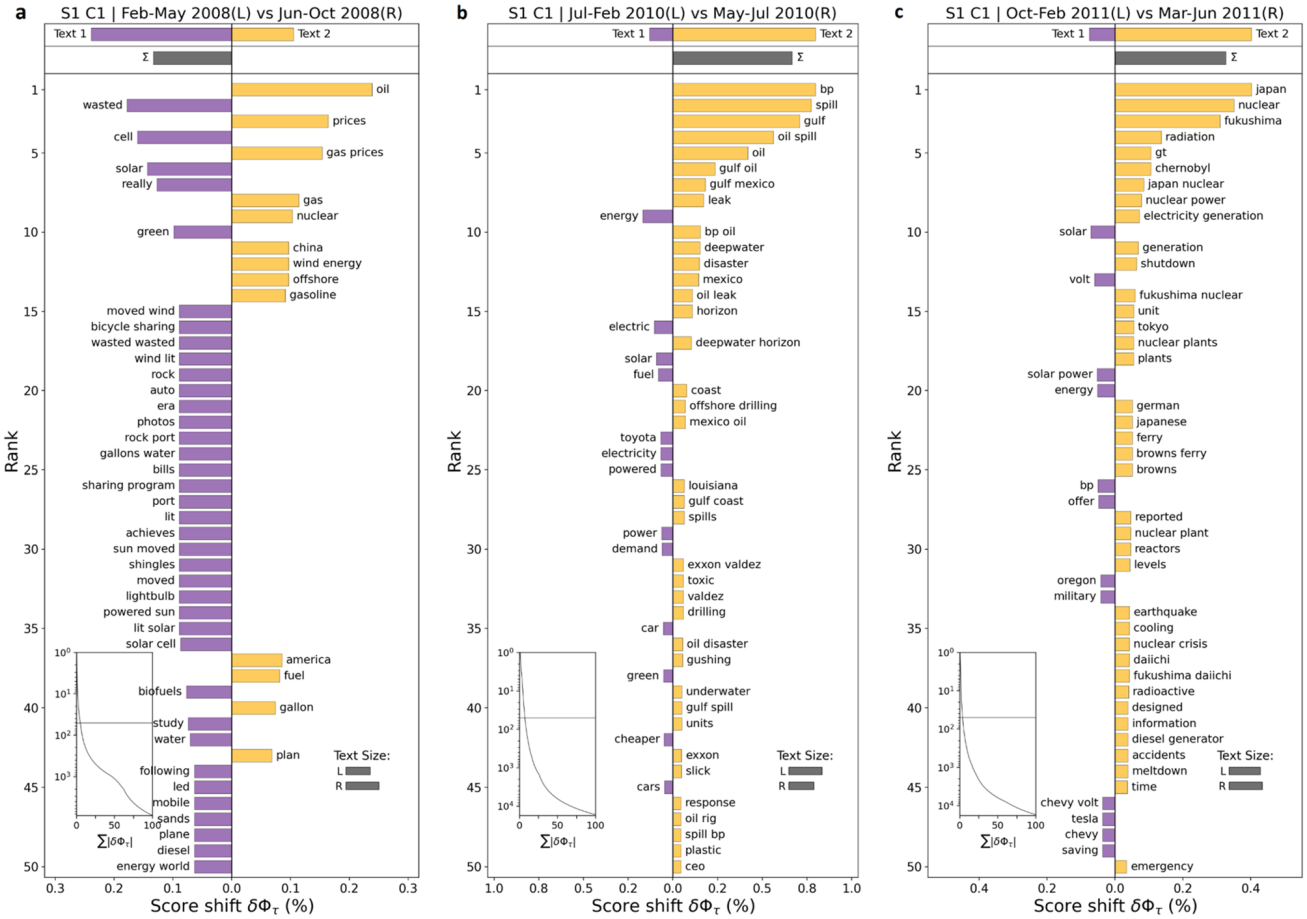
Word shift graphs for *Energy* cluster in Sample 1 showing the texts from the time periods with unusually high vs. low user activity. A deeper look at the underlying keywords from the respective time periods of unusually high activity reveals that the discussions within the *Energy* cluster spiked in 2008 due to *oil price shock* (**a**), in 2010 due to the *Deepwater Horizon oil spill* (**b**), and in 2011 due to the *Fukushima nuclear disaster* (**c**).

In the *Energy* cluster, we observe that overall the discussions mainly spiked three times (Fig. 9a) since 2008; twice due to oil related events—(1) oil price shock^70^ in 2008 (Fig. 10a, Extended Data Figs. S3a,f, S4a) and (2) Deepwater Horizon oil spill^71^ in 2010 (Fig. 10b, Extended Data Figs. S3a,f, S4b)—and third time in 2011 due to Fukushima nuclear disaster^72^ (Fig. 10c, Extended Data Figs. S3a,e, S4c). Apart from that, we observe a somewhat gradual decline in the discussions within Energy cluster since 2013 implying that the focus of the public has relatively shifted downwards in *Energy* in general and has moved on to other climate related themes over the last decade, and it largely spikes during major energy related disasters and shock events.

Observing the monthly time-series of proportion of sub-clusters within the *Energy* cluster (Extended Data Fig. S3) further reveal that apart from energy related shocks, the public also responds strongly to scientific breakthroughs and climate related government initiatives. For instance, we observe an unusual spike in the *Alternative energy resources* sub-cluster, towards the end of December 2008 (Extended Data Fig. S3b), mainly associated with *the world’s first biofuel powered flight test in New Zealand*^73^ (Extended Data Fig. S5a,b)*.* Further, we observe that there is a gradual decline in the discussions related to *electric vehicles and alternative energy resources* from 2008 to 2014, however after that it starts to increase gradually again reaching another peak in 2020. We note that while during 2017 the discussions mostly relate to the comparison between *internal combustion engines* and *electric vehicles,* in 2020 the focus seems to have shifted towards *fuel cell vehicles* and *building better infrastructure for batteries* in general (Extended Data Fig. S5c). Furthermore, in the *Solar energy* sub-cluster, we are able to identify three clear spikes in discussions; mainly due to (1) the announcement of *the EU renewable energy directive 2009/28/EC*^74^ in 2009 (Extended Data Fig. S6a), (2) the approval by the US for *the world’s largest solar energy project*^75^ to be built in California in 2010 (Extended Data Fig. S6b) and, (3) the report on *Trends in Photovoltaic Applications*^76^ published by the International Energy Agency (IEA) in 2015 implicitly stating that the Australian rooftop solar energy is among the cheapest in the world (Extended Data Fig. S6c). Apart from that, the monthly proportion of discussions remain mostly within the ± 1 standard deviation for the rest of the time period.

In the *Renewable energy excl. solar* sub-cluster also, we observe that mostly the discussions remain within the ± 1 standard deviations over the entire study period except for two sharp drops during 2008 and 2010—which coincide with the shift in the discussions towards *oil price shock (2008)* and *Deepwater Horizon oil spill (2010)*—and a rise in 2017 which was caused majorly by the combination of the announcements of the *France’s climate plan*^77^ (Extended Data Fig. S7b) and *the public water supplies (Scotland) amendment regulations*^78^ (Extended Data Fig. S7a) during the year. In the *Nuclear energy* sub-cluster, on the other hand, we observe that apart from the *Fukushima nuclear disaster* in 2011, another spike in discussions was observed during 2013 again related to the same event but for different reason—mainly because the *Fukushima’s radioactive water leak*^79^ was revealed (Extended Data Fig. S7c). Also after 2013, we observe that the proportion of discussions related to *Nuclear energy* in general stays above the historical mean, with only occasional downward movements in between, implying that *nuclear energy* stays as one of the key topics of discussion on Reddit over time. Finally, within the *Oil & gas* sub-cluster, we observe that in addition to *Oil price shock* in 2008 and *Deepwater Horizon oil spill* in 2010, the discussions remained mostly above historical average from 2011 to 2015 (Extended Data Fig. S3f). Although, a sudden decline was observed in the discussions related to *oil & gas* beginning from 2016 and it mostly stayed below the historical mean thereafter with only breaking out slightly above it in 2020 for a small period of time before it began to fall again.

All in all, we note that a variety of sub-themes comprise the discussions within *Energy* cluster, and all the 5 sub-clusters identified within it have different underlying features that influence the overall discussions related to the broader theme *Energy* on Reddit.

**Figure 11:**
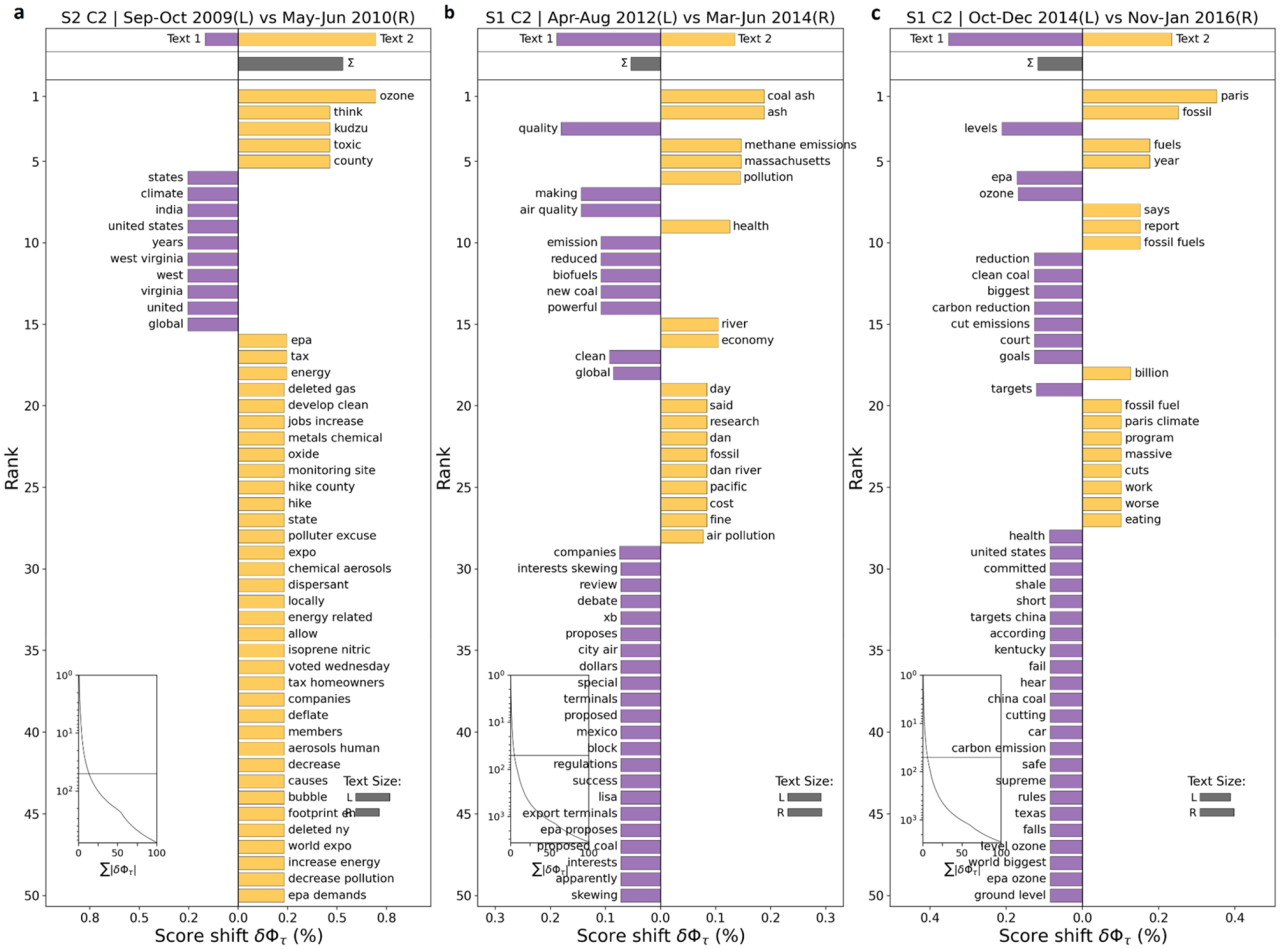
Word shift graphs for *Carbon [emissions]* cluster showing the texts from the time periods with unusually high vs. low user activity. The discussions within the *Carbon [emissions]* cluster spiked in 2009 mainly as West Virginia passed the *Alternative and Renewable Energy Portfolio Act* and US-India launched *the Partnership to Advance Clean Energy (PACE)* (**a**). In 2014, the rise was attributable to Dan *river coal ash spill* and a report on *high methane emissions from natural gas infrastructure in Massachusetts* (**b**). Another peak was observed towards the end of 2015 mainly due to the discussions around *The Paris Agreement* of 2015 (**c**).

In the *Carbon [emissions]* cluster, we observe that the discussions usually keep on fluctuating around the historical mean and majorly spike only during key climate related events (Fig. 9b). In 2009, we see that the monthly proportion of Reddit users’ discussions breaks above the + 1 standard deviation, partially as West Virginia passes *the Alternative and Renewable Energy Portfolio Act*^80^ in the month of June to reduce dependence on coal requiring the utilities with over 30,000 residential customers to get 25% electricity from renewables by 2025. It was further supported by the launch of the US-India *Partnership to Advance Clean Energy (PACE)*^81^ in the month of November to accelerate inclusive low carbon growth with advancements in new clean energy technologies and supporting research (Fig. 11a). After 2009, we note that the discussions related to carbon [emissions] remained somewhat subdued until 2014 when, due to a drainage pipe burst in North Carolina, 39,000 tons of *coal ash got spilled into the Dan river*^82^. In addition, we observe that users had concerns related to high methane emissions being released from natural gas infrastructure^83^ in Boston, Massachusetts in 2014 (Fig. 11b). We observe a sudden fall in discussions after that for a small period of time until it starts to rise again and reaches another peak towards the end of Dec. 2015, when users' focus shifted considerably towards the *Paris Agreement of 2015*^84^ (Fig. 11c). Apart from that, the discussions mostly remained within the one standard deviations except for some occasional spikes around *California wildfires*^85,86^ (2017–2018), *Australian bushfires*^87^ (2019–2020) and the announcements of the pledges for *Net zero emissions by 2050*^88^ by majority of the countries globally (2021)*.*

In the *Administration* cluster, we see that the discussions are mostly below the historical mean in the initial period starting from 2008 (Fig. 9c) with only occasional rises above mean with respect to some key climate related events such as *Copenhagen Climate Change Conference* in December 2009^89^. However, we see a sharp rise in the discussions breaking out above + 1 standard deviation in 2012 during US presidential elections (Extended Data Fig. S8a), mainly as the public was disquieted by the *lack of emphasis on climate* by both the candidates in the election debates^90,91^, so much so that a new term *“Climate silence”* was coined in response. Moreover, the Republican nominee Mitt Romney, was particularly in focus as he claimed to be uncertain about the *man-made impacts* on global warming^92^.

After 2012, we observe a sharp decline in the discussions and they remain mostly subdued until 2017, when a series of climate related unpopular actions and policy changes taken by Donald Trump’s administration^93^ coerced the climate activists to react and be more vocal about the climate change issues resulting in a nationwide protest in the US, also known as *2017 People’s climate march*^94^, to oppose the environmental policies of Donald Trump and his administration (Extended Data Fig. S8b). We also note that the monthly proportion of discussions remain mostly above historical mean beyond 2017 with *climate* remaining as one of the key areas of concerns from *administration* perspective on Reddit.

**Figure 12:**
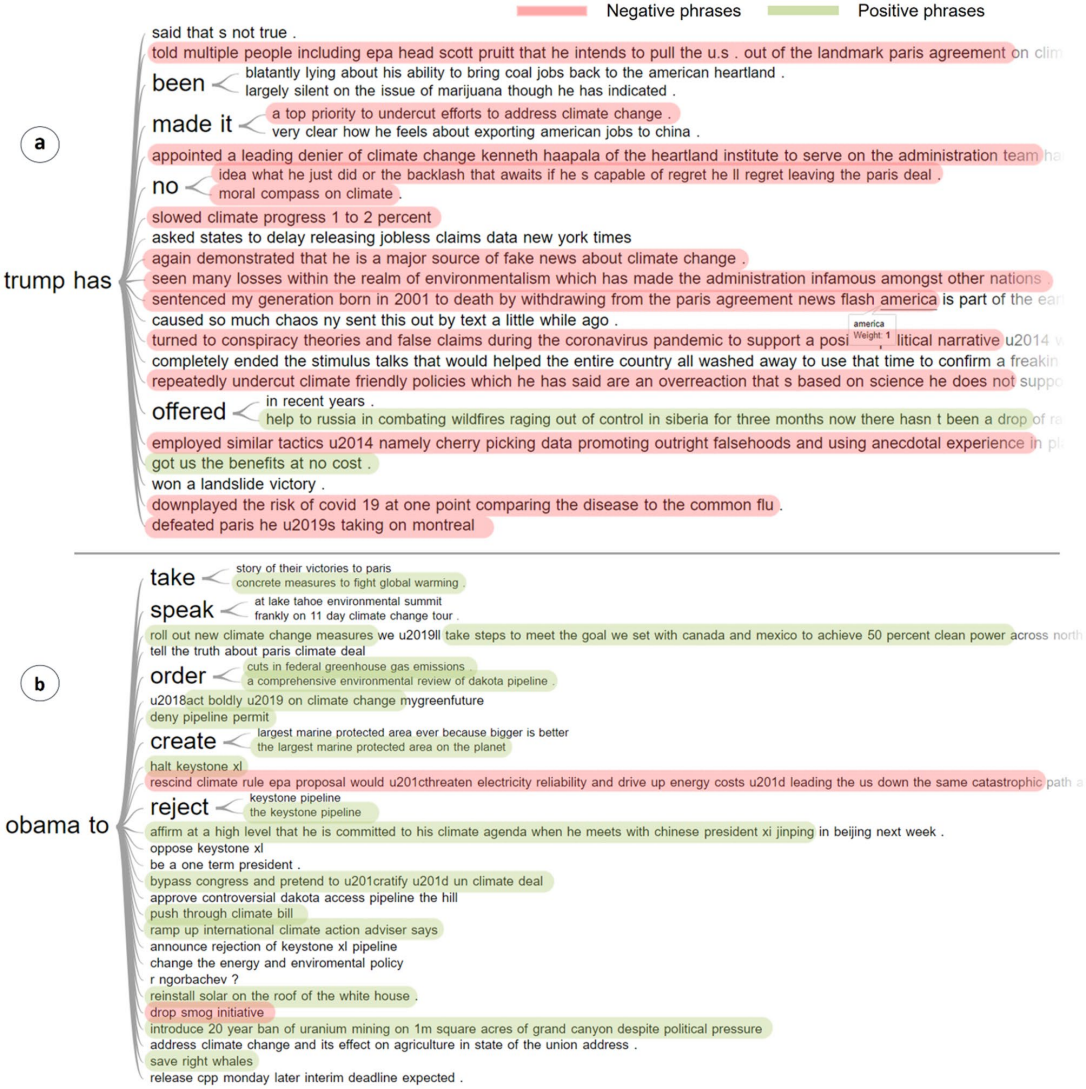
Word trees generated from the *Administration* cluster comparing a set of random phrases from discussions related to the former US presidents—Donald Trump and Barack Obama. The phrases with positive sentiments are highlighted with *green* colour and the phrases with negative sentiments are highlighted with *red.* A stark contrast can be observed in the expressed sentiments of the Reddit users when comparing the discussions related to the former two US presidents. People sound highly dejected when it comes to discussions related to *Donald Trump’s* policies and actions on climate change, and so expressed mostly negative sentiments as a consequence (**a**). However, they were more accepting when it came to discussions related to *Barack Obama’s* policies and actions, and so expressed mostly positive sentiments as a result (**b**).

Furthermore, we generate word trees from the text phrases collected from the *Administration* cluster to visualize the discussions interactively (Fig. 12). Interestingly, by comparing the discussions related to *Donald Trump* and *Barack Obama,* we observe that the public was highly discontented with Trump's handling of environmental policies on multiple fronts, and exactly in opposite, was more accepting of the approach taken by Obama. As Trump initiated a broad rollback of Obama’s environmental policies after assuming office^95^, the discussions were mostly associated with negative sentiments and disquiet, as highlighted with *red* colour in the figure. On the other hand, in case of Obama, we note two key points; (1) the average level of monthly proportion of discussions was significantly lower in Obama’s administration as compared to Trump’s administration, implying that the public in general was less concerned with *administrative actions and policies* from *climate change* perspective during that time, (2) Obama took a number of initiatives to tackle *climate change*, and so most of the discussions were associated with positive sentiments, as highlighted with *green* colour.

In the *Climate science* cluster, we observe that discussions rose sharply beginning from 2008 (Fig. 9d), reaching the highest peak towards the end of 2009 with the *“climategate” scandal*^96^ in November followed by the *Copenhagen Climate Change Conference*^89^ in December (Extended Data Fig. S8c) and falling sharply thereafter. After 2009, the discussions remained mostly below the historical mean until Sept. 2013 when IPCC (Intergovernmental Panel on Climate Change) issued *its starkest warning over global warming*^97^ and held man-made activities such as burning of fossil fuels and deforestation responsible (Extended Data Fig. S9a). Another peak in discussions is observed in the same month next year mainly due to *2014 People’s climate march*^98^*,* when a large number of people began organizing in September in response to the then UN Secretary-General Ban Ki-moon’s call to global leaders to gather at the *2014 UN Climate Summit*^99^ (Extended Data Fig. S9b). Apart from that, except for a spike around *the Paris Agreement of 2015*^84^*,* the discussions mostly keep on fluctuating around the historical mean till end 2019 and register a sharp drop at the beginning of Covid-19^100^ pandemic reaching a new low in a decade. Further, we identify a few of the key underlying themes within the cluster using word trees (Fig. 14a,c, Extended Data Fig. S13). We note that some of the key areas of *debate* within the *Climate science* cluster broadly relate to climate change, climate science, man-made global warming and divestment from fossil fuels to combat climate change. However, the *denial* of climate science and climate change is condemned by the Reddit users in general. Additionally, we observe a lot of discussions specifically regarding the *“change”* in *climate,* particularly in the context of it “happening in the present or has already happened” with extremely negative sentiments associated with it. Also, when we searched the keyword *“study shows”* within the cluster, it was mostly followed by phrases with negative sentiments associated with it.

**Figure 13:**
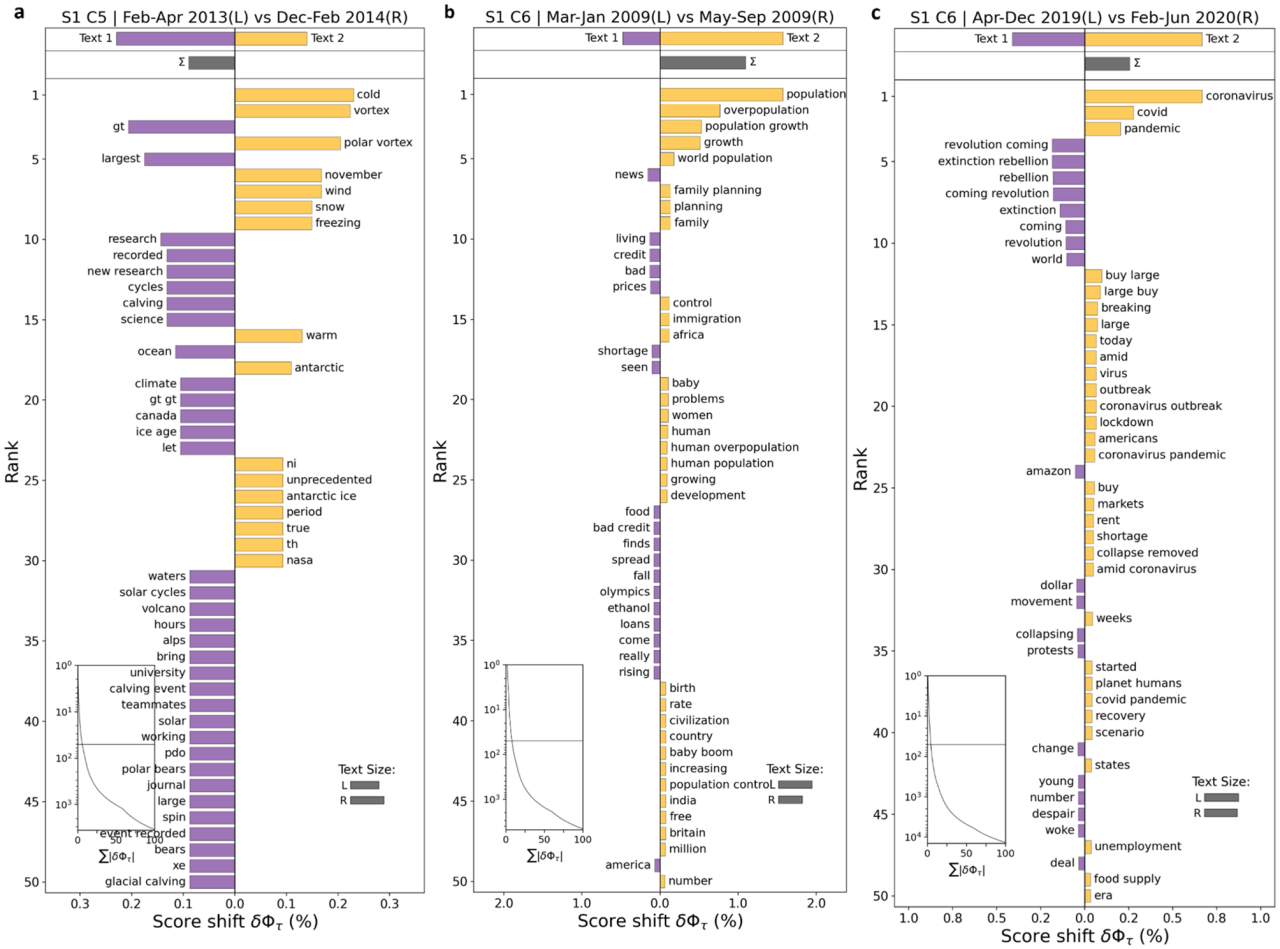
Word shift graphs for *Global warming* (**a**) and *Population & economy* (**b**, **c**) clusters showing the texts from the time periods with unusually high vs. low user activity. The discussions within the *Global warming* cluster spiked in 2014 mainly as the *early North American cold wave*, also known as *“polar vortex”*, hit parts of Canada and parts of the US (**a**). In the *Population & economy* cluster, we see a sharp rise in discussions beginning in early 2009 reaching an all-time high peak in the month of July with the publication of the *State of World Population 2009* report by the UN (**b**). Another peak within the cluster was observed in 2020 as the focus of the discussions shifted towards *Covid-19* (**c**).

In the case of the Global *warming* cluster, we observe a significant volatility in the discussions over the entire study period (Fig. 9e), implying that it continues to be a key theme across discussions and over time. In general, there is a gradual rising underlying trend in discussions related to *global warming* beginning from 2008 and reaching the highest peak in Jan. 2014 as the *early North American cold wave*^101^, also known as *“polar vortex”*, hit parts of Canada and parts of the north-central and upper eastern US (Fig. 13a). After that however, it starts to show a gradual declining underlying trend, though hovering around the historical mean in general.

**Figure 14:**
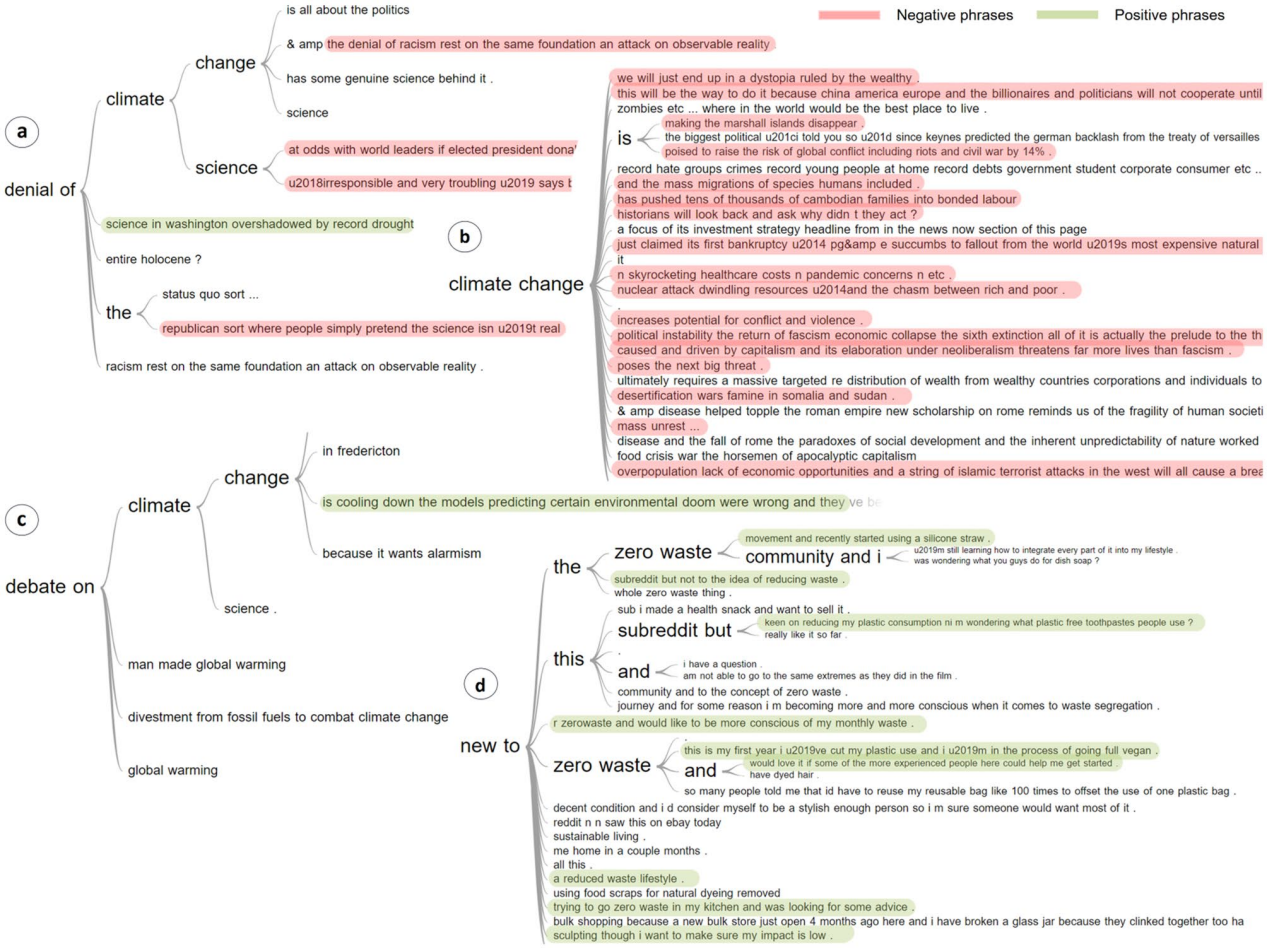
Word trees generated from a set of random phrases taken from *Climate science, Population & economy,* and *Plastic & waste* clusters. The plots (**a**, **c**) show the phrases from the *Climate science* cluster with the user discussions around the *denial* and *debate* on climate science, climate change, and man-made global warming etc. in general. In case of the *Population & economy* cluster (**b**), we observe a lot of *concerns* around *climate change* including the increase in potential for conflicts and violence, dwindling resources etc*.*, calling it *the next big threat*. Lastly, in the case of the *Plastic & waste* cluster (**d**), we observe a lot of discussions around *zero waste* wherein many Reddit users claimed to be new to the concept.

In the *Population & economy* cluster, we see a sharp rise in discussions beginning in early 2009 and reaching an all-time high peak in the month of July (Fig. 9f) with the focus of Reddit users suddenly shifting towards overpopulation, population growth, family planning etc*.* (Fig. 13b). It was mainly due to the publication of the *State of World Population 2009* report^102^ by the UN, wherein it emphasized on the shift from abstract technical discussion about countries’ carbon emissions to human beings, world population and women with a focus on family planning in particular. However, after that period, we observe that the discussions related to population and economy in general mostly keep on hovering around the historical mean within ± 1 standard deviations reaching a relatively smaller peak in 2020, with the focus of the discussions shifting towards *Covid-19*^100^ (Fig. 13c), before falling sharply again thereafter. From the climate change perspective in general we observe one of the key areas of concern remains around the rising *income inequality* in most countries over the past two decades^103^ (Fig. 14b). Apart from that, the risks of global conflicts including riots and civil wars, mass migration of species including humans, financial implications of natural disasters, overpopulation, skyrocketing healthcare costs, dwindling resources and political instability remain the other major areas of concerns.

The *Plastic & waste* cluster had by far the most unique profile of the distribution of the monthly proportion of discussions over time (Fig. 9g). With only a small peak at the beginning of 2008, coupled with a decade-long period of relatively muted discussions, plastic and waste did not seem to be a central theme in the climate related talks at all. However, it all changed considerably in June 2018 when Norway submitted a proposal to amend the *Basel Convention* to specifically include plastic waste under its purview^104^. Subsequently, the discussions reached another all-time high in May 2019 when Basel Convention finally adopted the *Plastic waste amendments*^105^. Notably, the level of the discussions still stays far above the pre-2018 level implying the high level of influence that international waste management policies such as *Basel Convention* hold in dictating the public discussions on plastics and waste in general. In fact, it was observed that after the adoption of *Plastic waste amendments* a lot of people on Reddit started inquiring about *“zero waste”* and reducing *“waste”* (Extended Data Fig. S9c), claiming that they were new to the zero-waste concept or community, and a reduced waste lifestyle in general (Fig. 14d).

In the case of the Agriculture *[and administration for Sample 1]* cluster, although the time series for the two samples differ slightly in terms of underlying content—with administration related discussions in Sample 1—we observe a similar underlying trend with discussions gradually declining over the entire study period (Fig. 9h). Here, one of the key policy events that led to much discontent among the Reddit users was the inclusion of the *Section* *735* in the Consolidated and Further Continuing Appropriations Act, 2013^106^, also dubbed as “*The Monsanto Protection Act”*^107^, by the Obama administration (Extended Data Fig. S10a), allowing the farmers to continue the cultivation and commercialization of the crops (o/w non-regulated status has been invalidated), while the legal challenges related to the safety of those crops would still be pending. This apparently was also the time period when the discussions reached its peak within this cluster. The discussions related to “monsanto protection act” or even “monsanto” in particular were associated with extreme negative sentiments, as the users blamed the Monsanto Company for corruption and escaping regulations (Extended Data Fig. S14). Apart from that, we observe a lot of discussions around genetically modified/engineered crops/foods etc.

In the Wildlife cluster, we observe a sharp drop in the discussions initially in June 2008 before it quickly climbed back up in 2009 (Fig. 9i) mainly as; (1) a large population of rare Irrawaddy dolphins was discovered in Bangladesh^108^, (2) a study revealed that the Irrawaddy dolphins from the Mekong river, Cambodia were in danger of disappearing due to high pollution in the river^109^ and, (3) the 2009 H1N1 pandemic^110^ broke out in Mexico and subsequently spread to the other countries around the world including the US (Extended Data Fig. S10b). Moving forward, the discussions within the cluster keep on hovering around this elevated level for a prolonged period till registering a sudden fall in 2017. However, post that, after a period of around three years of low activity, the discussions started to climb again, gradually towards the end of 2019 mainly as users started to show concerns in regard to Australian bushfires^111^**.** The discussions reached another peak in 2021 reclaiming its pre-2017 level as the users voiced concerns over; (1) the volcanic eruption in Congo^112^ in May and, (2) a new study on *North Atlantic right whales*^113^ in June, stating vessel strikes and entanglement in fishing gear as key threats to their growth (Extended Data Fig. S10c). We observe that broadly the discussions relate to a wide range of “critically endangered” species, extinction crisis, and its causes (Extended Data Fig. S15).

The *Natural catastrophe* cluster, as its name implies, mainly observed heightened user activity during the natural disasters and related events. We note that the proportion of discussions in this cluster started at a very low level in 2008 (almost negligible), and stayed at that level until two severe earthquakes^114,115^ hit Japan and New Zealand in 2011 (Fig. 9j). Beyond that period, the discussions remain relatively elevated on an average as compared to the pre-2011 levels, implying that the public’s interest in the *natural catastrophe* theme has increased in general over time. It reached the highest peak in 2017 when a series of hurricanes namely Harvey^116^, Irma^117^ and Maria^118^ made landfall one after the other, wreaking havoc in and around the areas in the North Atlantic ocean (Extended Data Fig. S11a). Another major spike in discussions is observed exactly a year after in Sept. 2018, mainly as hurricane Florence^119^, typhoon Jebi^120^ and typhoon Mangkhut^121^ made landfall in and around the areas of US, Japan and southeast Asia respectively (Extended Data Fig. S11b). Additionally, on a broader level, we note that most of the studies related to natural catastrophes and climate change are associated with negative sentiments over time, and the public in general showed great concerns over rising frequency and intensity of the disasters (Extended Data Fig. S16).

In the *General posts* cluster, the discussions mostly hover around historical mean till 2016 (Fig. 9k), except for a significant peak in 2012 mainly associated with the discussions related to *Rio*+*20*, UN Conference on Sustainable Development^122^ (Extended Data Fig. S11c). Also, beyond 2016 we observe a gradual rise in the general climate related discussions overall. Finally, in the case of the *Unidentifiable* cluster, although we observe a gradual rise in discussions over time, with peaks in 2016 and 2021 (Fig. 9l), we were unable to identify any key climate related events responsible for those movements in the discussions within this cluster (Extended Data Fig. S12a,b).

### Optimum clusters full order partial correlation analysis

**Figure 15:**
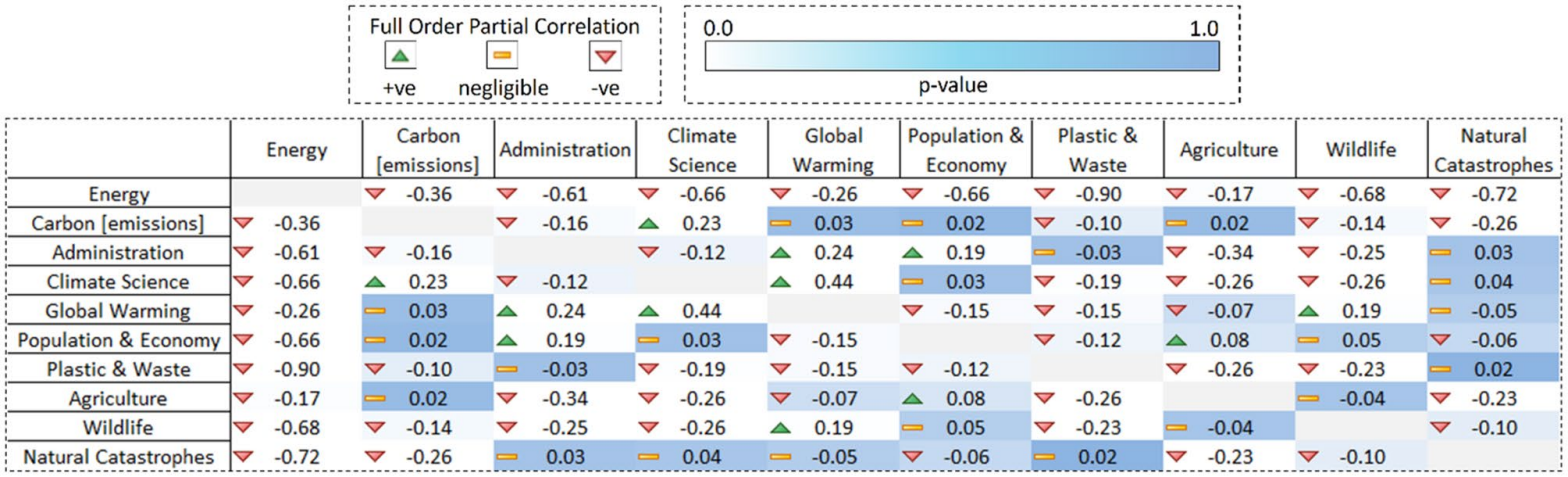
Optimum clusters full order partial correlation matrix with *p* values. The figure (**a**) shows the full order partial correlations for 10 unique climate change related themes as identified. The up arrow in green signifies a positive partial correlation between the two clusters whereas the down arrow in red signifies a negative partial correlation between the pair. The background colour of the cell represents the *p* value for the respective partial correlation of a cluster pair ranging from 0 (in white colour) to 1 (in blue colour).

In order to measure the strength of the relationship between the 10 identified unique climate related themes, we perform a full order partial correlation analysis on the time series output from previous step, and provide the results in the form of a matrix (Fig. 15). We observe statistically significant relationships in a number of theme pairs with varying range of full order partial correlation values. In the case of *Energy* theme, as majority of the discussions relate to renewable sources (c.64%) which usually alleviate climate change concerns rather than aggravating them^123^, we see an overall negative relationship with the rest of the climate related themes. *Plastic & waste* has the strongest negative correlation with *Energy*, indicating that as the discussions related to the former theme go up, the focus of the discussions shifts significantly from the latter theme and vice versa. In case of *Carbon [emissions]*, we observe a statistically significant negative relationship with *Administration, Wildlife,* and *Natural catastrophes*, as opposed to a positive relationship with *Climate science* thereby indicating that as the discussions related to climate science theme go up, the focus of the discussions also shifts towards carbon [emissions] and vice versa. Notably, we observe no statistical significant linear relationship between *Carbon [emissions]* and *Global warming* related discussions.

In case of Administration, we observe a statistically significant positive relationship with *Global warming* and *Population & economy*, thereby indicating that discussions related to administration, politics etc. also encourages discussions in relation to rising global temperature, over population, and economic health etc. Also, a slightly negative partial correlation with *Agriculture* and *Wildlife* indicates that the rise in administration related discussions also shifts the focus of the discussions from agriculture and wildlife related themes and vice versa. When it comes to *Climate science*, we observe a statistically significant positive relationship with both *Carbon [emissions]* & *Global warming*, and a negative relationship with *Plastic & waste, Agriculture,* and *Wildlife* thereby showing that when discussions related to climate science rise, the focus of the discussions mostly remains on emissions, global temperature, new studies etc. and is comparatively less on food, waste, farmers, wildlife conservation etc.

Apart from that, we also found a statistically significant positive relationship between *Global warming* and *Wildlife*, thereby showing that concerns related to rising global temperature, sea levels etc., and forests, life, extinction etc. are positively associated by the public. Also, we observe slightly negative partial correlations between *Global warming*, and both *Population & economy* and *Plastic & waste* themes, mainly indicating that public does not associate global warming with overpopulation, economic health or zero waste etc. in general. In the case of *Population & economy*, we also observe a slight negative relationship with *Plastic & waste*, thereby showing that as the concerns related to population growth, financial crisis etc. rise, public’s focus tends to shift from sustainability, low waste themes etc. and vice versa.

We also observe slightly negative statistically significant relationship between *Plastic & waste* and both *Agriculture* & *Wildlife* themes, which simply indicates that concerns related to plastic, waste, sustainability etc. go down as the public has more discussion on agriculture and wildlife related themes. Finally, we observe a statistically significant negative relationship between *Natural catastrophes* and *Agriculture* thereby indicating that in the event of a natural disaster, public tends to focus less on food, farming etc. and vice versa.

## Discussions and conclusion

We develop a machine learning based approach to identify, store and process climate related posts on the social media platform, Reddit. Using USE, a state-of-the-art sentence encoder, and K-means clustering algorithm allows us to classify the climate related posts automatically, at a scale, and without any human intervention. Further, training a Random forest based binary classifier for all of the optimized clusters separately helps us in identifying the key underlying themes comprising the climate related discussions on Reddit since its inception. The results from the clustering application allow us to not only identify the major themes related to climate change, but also detect novel trends within, and follow them through time. Finally, performing a full order partial correlation analysis on the identified themes helps us in answering some of the key questions such as; if there exists a relationship between various climate related themes? And if yes, how do they influence each other?

Overall, we identify that there are broadly 10 distinct underlying themes comprising the climate related discussions on Reddit. Notably, the distribution of themes is not well diversified as some themes such as *Energy, Wildlife* and *Climate science* get considerably more traction as compared to the other themes such as *Carbon [emissions], Administration, Global warming, Population & economy, Agriculture, Plastic & waste* and *Natural catastrophes*. Also, by comparing the results of the two random and distinct non-overlapping samples, it is realized that broadly the underlying themes remain the same over time with only slight differences in terms of their construction and composition. Finally, a full order partial correlation analysis revealed some of the key statistically significant relationships among different climate related themes, which could particularly be appealing to the decision makers.

For instance, in the case of *Administration* theme, we observe a statistically significant positive relationship with *Global warming* and *Population & economy* themes, whereas a statistically significant negative relationship with *Carbon [emissions]*, *Agriculture,* and *Wildlife* themes. Thus, it mainly indicates that although administration related discussions in general have a positive influence on the discussions in relation to global warming, overpopulation, and economic health etc. on social media, it has not been much successful in communicating the concerns in relation to rising carbon [emissions], sustainable farming, or wildlife conservation etc. to the general public. It shows a clear gap in the public communication by the administration especially when we compare this observation with a couple of the key goals embedded in the Paris Agreement^124^. While the administration has been successful in positively influencing discussions on global warming (an outcome of climate change) in line with the Paris Agreement goal of limiting global temperature increase to well below 2 degrees Celsius^125^, it has failed to communicate well on the primary driver of the same i.e. carbon emissions^126^ (a cause of climate change), which relates to another important Paris Agreement goal of reaching global peaking of greenhouse gas emissions (GHGs) as soon as possible^124^. In simple words, the administration has successfully communicated the effect of climate change to the public so far, but it failed to appropriately communicate the underlying reason that is causing that change in the first place. However, a clear targeted communication from administration is necessary to spread awareness among the public about the causes of climate change even more so than the effects of it, if the administration expects to have a collective desired response from public in the fight against climate change. For example, in the case of *Climate science* theme, we observe a statistically significant positive relationship with both *Carbon [emissions]* (cause) and *Global warming* (effect) themes thereby implying that climate science is doing a relatively better job than administration in influencing the discussions in the right direction in-line with the Paris Agreement. Apart from that, we also note that a rise in the administration related discussions tend to shift the focus of the public discussions away from topics such as energy, agriculture and wildlife related concerns. However, considering how significant role these themes play in the context of maintaining an overall equilibrium in the ecosystem^127–129^, decision makers should take appropriate actions to fill up the gaps in public communication in this space as well.

*Climate change* as such is a very broad topic, and encompasses a wide range of issues and perspectives, with an unprecedented growth in research in this area so much so that it has become too large to assess manually^130^. Further, it is not something that only a small group of people could influence by themselves. We need collective efforts from all the stakeholders including public, administration and academia alike to combat the complex challenges related to climate change on multiple fronts^131^. And since social media discussions act as a proxy for public opinions^132^, by mining and processing this valuable information, our approach is able to kill three birds with one stone; (1) within the broad theme of *climate change,* we narrow down the focus to 10 critical factors influencing the discussions on social media, (2) we propose an automated machine learning based approach to classify the large amount of ever growing text data, which would otherwise be impossible manually and, (3) since we are using social media discussions as our source of data, we are able to highlight key concerns from the perspective of the general public in the context of climate stakeholders.

With a few exceptions, comparability of data and replicability of results remain as some of the major limitations in social media studies^133^. Even if the methods have been clearly defined, most of the social media platforms have terms and conditions of usage which forbid the retention or sharing of the data collected from their platforms. However, our approach allows us to overcome this limitation, as we choose Reddit as our source of data, and with the help of Pushshift API, the same data as used in this study can be retrieved for free, and repeatedly over time if needed, thereby eliminating the data comparability and results replicability issues. Additionally, unlike other major social media platforms^15,134,135^ where the posts are largely random, Reddit platform is structured into theme based communities with well-defined rules when it comes to creating or sharing posts within those communities, and so the information is relatively rich in content, specific and more relevant as compared to other social media platforms. Furthermore, in the social media and climate change related studies, there is a limitation in terms of lengths of the study periods, with most of the studies^17–20^ focused on shorter time spans mainly due to restrictions and complexities associated with data collection and usage. However, Reddit, our chosen social media platform, does not impose those time bound restrictions, and the Pushshift Database can be queried to retrieve any post published in the past, even the first ever post published on Reddit back in Jan 2005, thus making it an ideal source for long-term social media studies.

Another limitation of our study is in the choice of the K-means clustering algorithm. Although, on the positive side, K-means is one of the most widely used clustering algorithm^136^ and we employ it in our study mainly because it is relatively easy to implement, scale and generalizes to clusters of different shapes and sizes. On the negative side, some of its biggest limitations include reliance on the user to specify number of clusters, high sensitivity to noise and outliers, and entrapments into local optima^137^. Although we try to minimize these impacts by incorporating human intervention at the second step of cluster optimization, we note that since it is a machine learning based approach, the results are subject to additional uncertainties. Furthermore, since we chose a full order partial correlation analysis to measure the strength of the relationship between different climate related themes, there is a possibility of false observations of negative correlation between different pairs.

Hence, one of the future scope of studies could be to explore some advanced methods^138^ such as regularised inverse covariance, Bayes nets or minimum partial correlation etc. for measuring the strength of relationship between climate related themes. Another future scope of studies could be to explore a variety of different machine learning or deep learning-based clustering algorithms to compare and contrast their performance on the same underlying dataset, and check if the optimization of the number of clusters and thereby underlying themes improves overall. Another future scope of studies could be the application of supervised learning^47^ algorithms on the output labelled data taken from the unsupervised learning algorithms. The trained model would be able to identify and classify the climate related posts on Reddit in real time, which could then be used to build live trackers that measure the level of engagement of the public with respect to identified driving forces on the go. This could be helpful to the various climate stakeholders, especially the policymakers, climate activists, and climate researchers, as they bridge the gap between academia and the public in general. While the focus of the scientific community stays on new discoveries and innovations, it is the policymakers, climate activists and researchers etc., who acknowledge these scientific studies at first, and then devise plans, strategies, awareness campaigns etc. that influence and spread awareness among the public. Furthermore, a comparison of the attributions of the climate related scientific literature^139^ with the attributions of the social media discussions in the future, could help reveal the exact reasons for gaps^140^ between scientific community’s delivery and public’s opinions, which could in turn help the various stakeholders in decision making to close the gap between the two sides.

All in all, our approach allows the climate stakeholders to have an additional tool in their arsenal which provides quantifiable insights from the unstructured text data taken from social media discussions and allows them to measure the social response to any key climate related event over time. It provides the exact underlying themes comprising the varying social responses to climate change events over time and narrows down the scope further, essentially to support targeted climate change efforts, as far as the public is concerned. For instance, if suddenly the level of discussions related to plastic and waste fall among the public, our approach could help identify the sudden shift in response and the underlying cause responsible for the same automatically without human intervention. This information could then be used by the various climate stakeholders in their decision-making process to define the focus, scope, and budget etc. of their efforts, thereby helping them with optimizing their capital and resources allocation, to achieve the best desirable social response possible.

## Supplementary Information

Supplementary Information.
