# Supplementary material for: Machine learning based attribution mapping of climate related discussions on social media
Source: Sci Rep. 2022 Nov 8;12:19033. doi: 10.1038/s41598-022-22034-1 (PMC9643343; doi:10.1038/s41598-022-22034-1)
Supplement: Supplementary file 1 — Supplementary Information. [file 41598_2022_22034_MOESM1_ESM.docx]

**Machine learning based attribution mapping of climate related public perceptions on social media**

**Appendix**

**
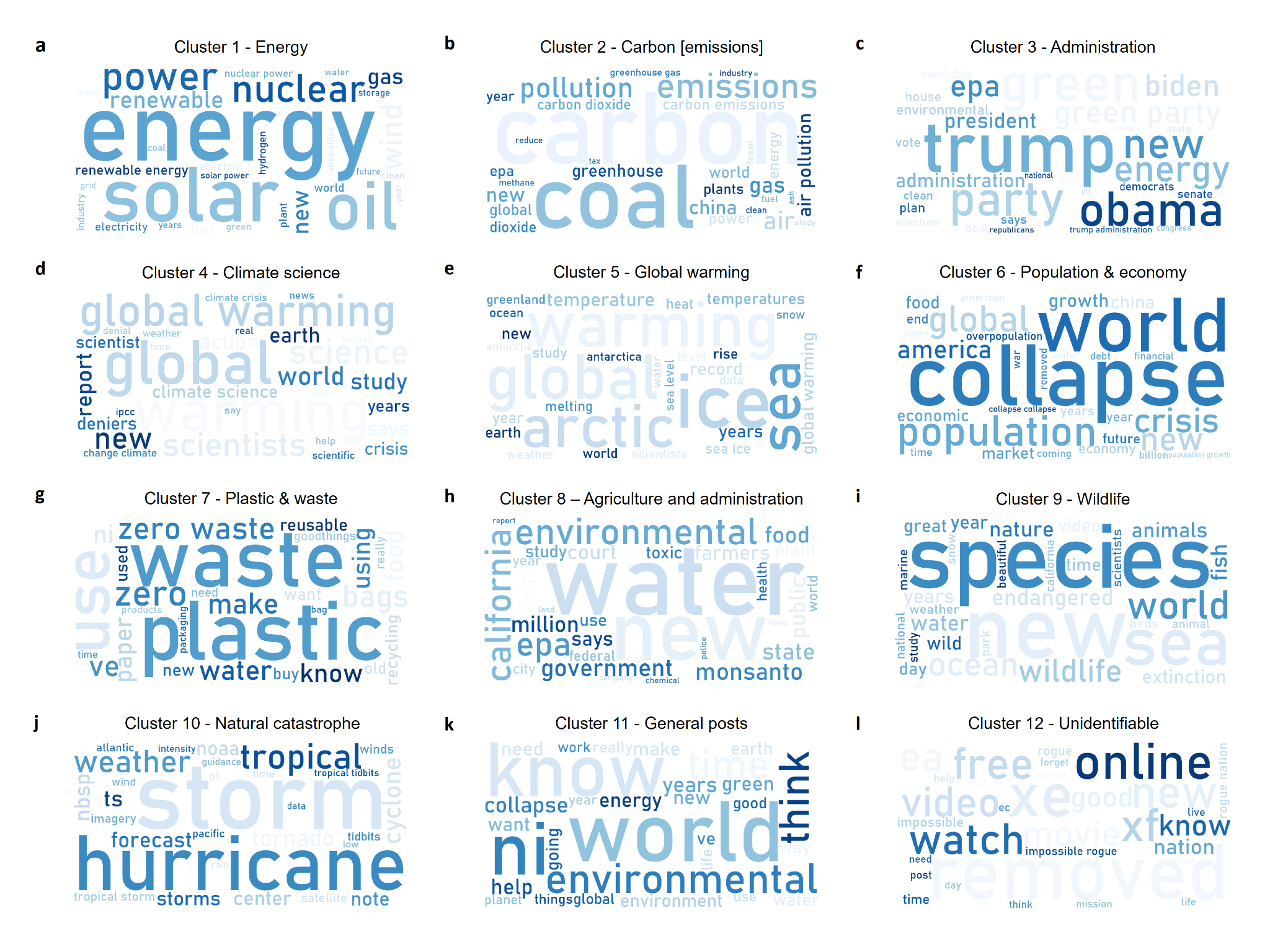
**

**Extended Data Fig. 1 | Sample 1 final optimized clusters in word clouds.** The word cloud for a given optimized cluster has been generated from top 30 keywords (unigrams or bigrams) featured in that cluster sorted by Tfidf-Vectorizer weights. It provides a bird view into the underlying keywords belonging to each cluster, specifically showing how distinguished the optimized clusters are in terms of their composition.

**
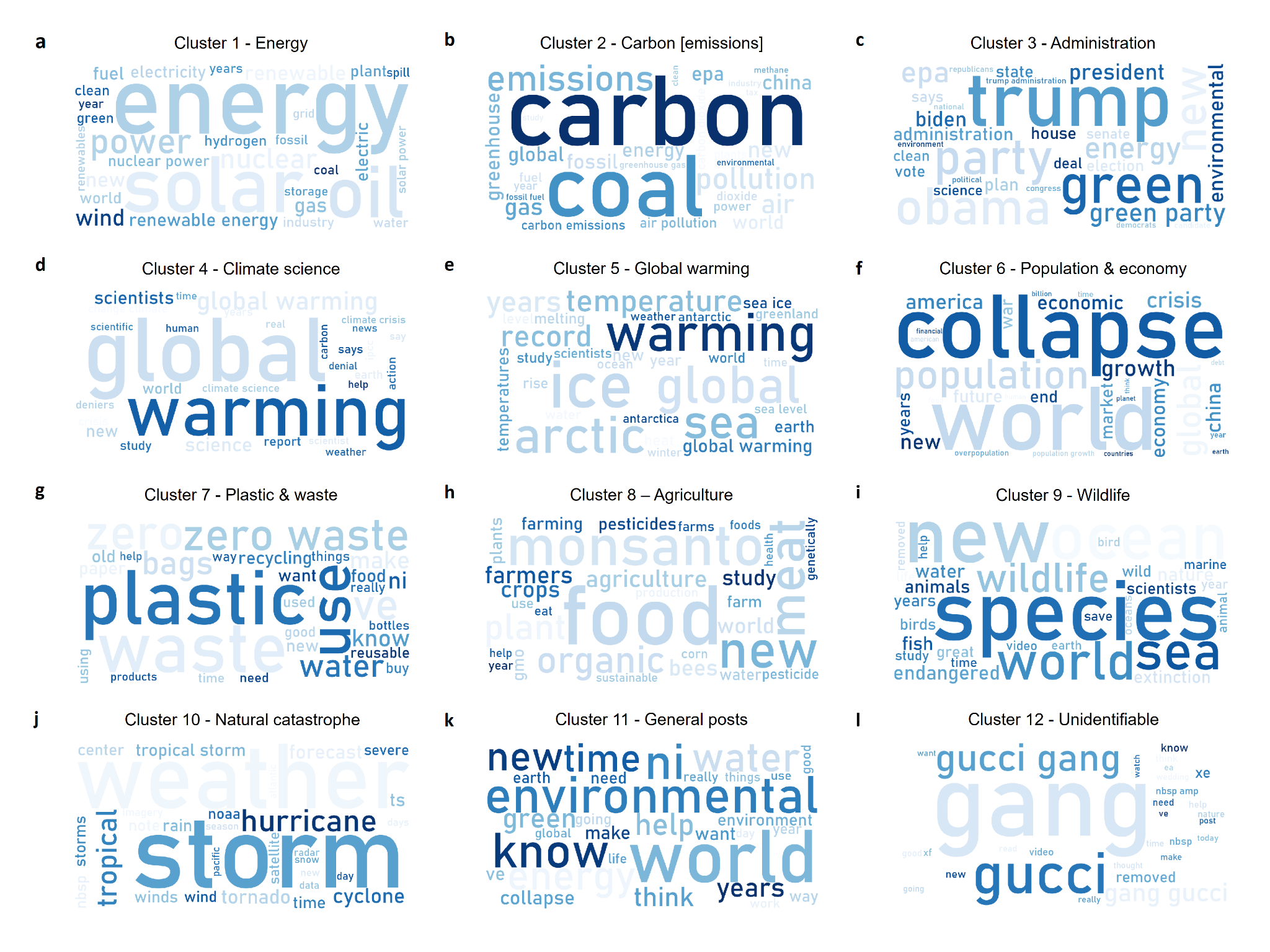
**

**Extended Data Fig. 2 | Sample 2 final optimized clusters in word clouds.** The word cloud for a given optimized cluster has been generated from top 30 keywords (unigrams or bigrams) featured in that cluster sorted by Tfidf-Vectorizer weights. It provides a bird view into the underlying keywords belonging to each cluster, specifically showing how distinguished the optimized clusters are in terms of their composition.

**
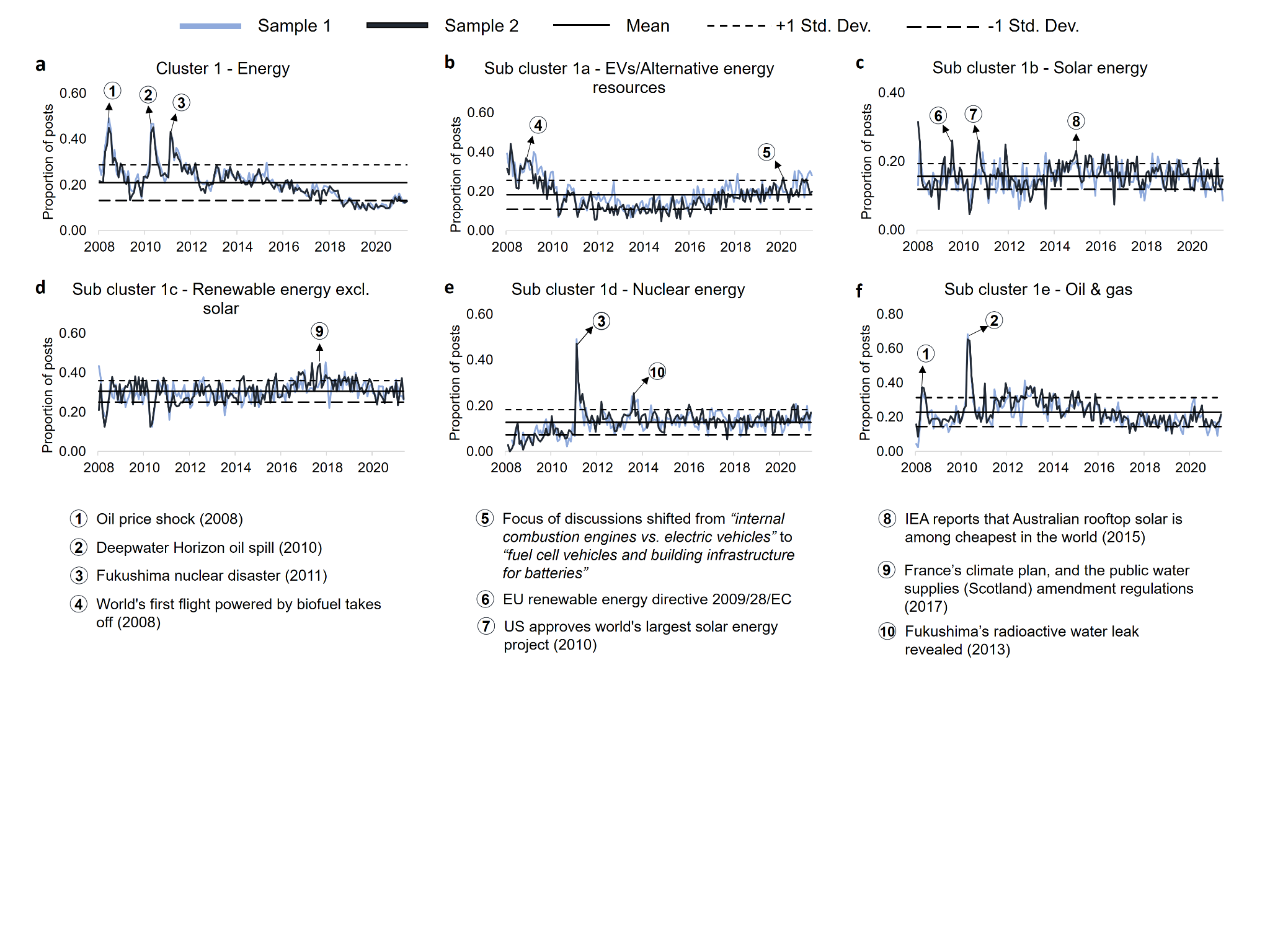
**

**Extended Data Fig. 3 | Monthly proportion of posts within the five identified sub-clusters of Energy (Cluster no. 1) over the entire study period 2008-21.** The figure shows the distribution of proportion of *Energy* sub-clusters on a monthly basis within sample 1 and 2 over the entire study period. The solid horizontal line depicts the overall mean of the sample 1 and 2 whereas the two dotted lines above and below depict the ±1 sample standard deviation for both the samples over the entire study period.


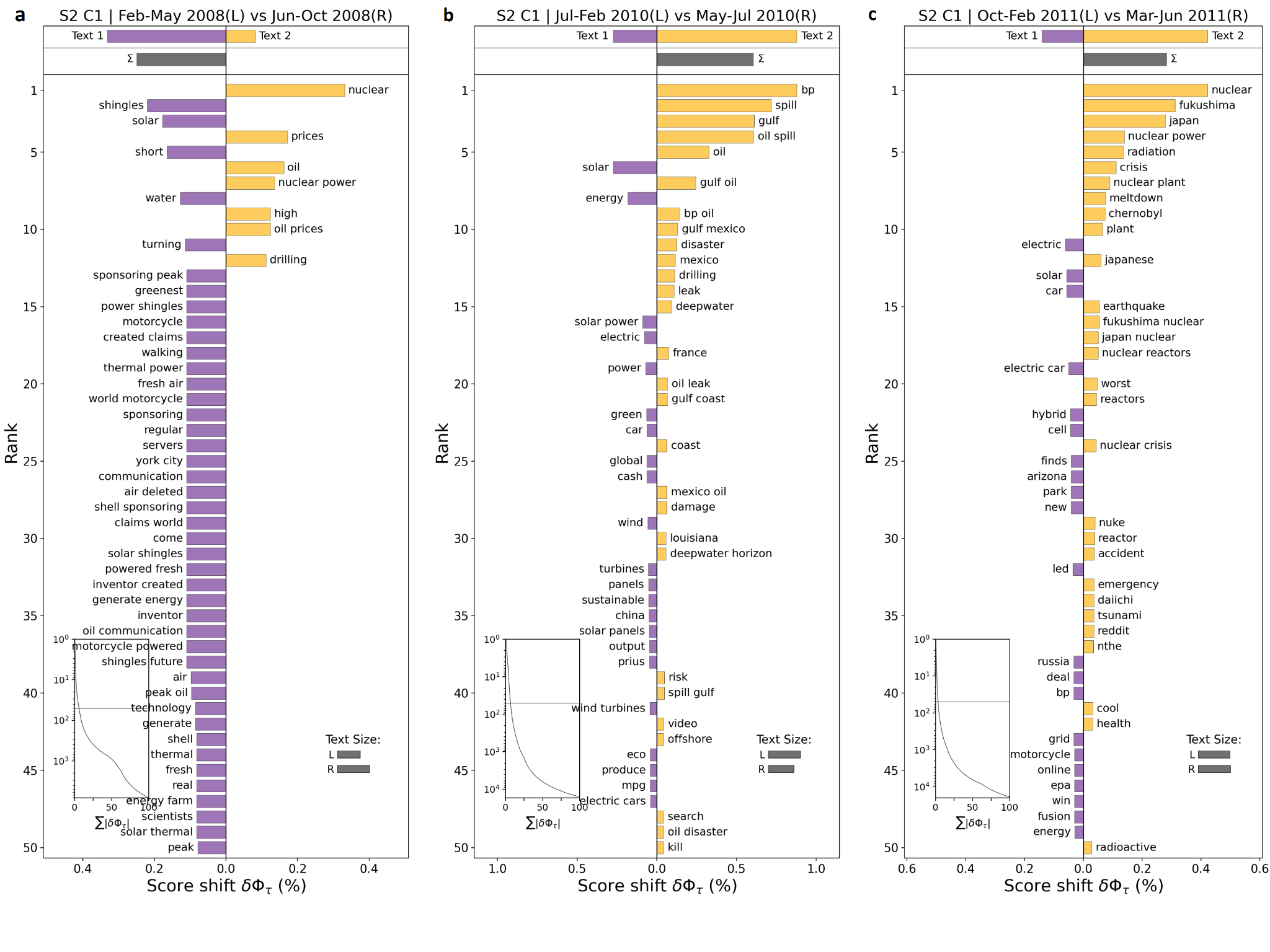


**Extended Data Fig. 4** **| Word shift graphs for *Energy* cluster in Sample 2 showing the texts from the time periods with unusually high user activity.** A deeper look at the underlying keywords from the respective time periods of unusually high activity reveals that the discussions within the *Energy* cluster spiked in 2008 due to *oil price shock* (**a**), in 2010 due to the *Deepwater Horizon oil spill* (**b**), and in 2011 due to the *Fukushima nuclear disaster* (**c**).


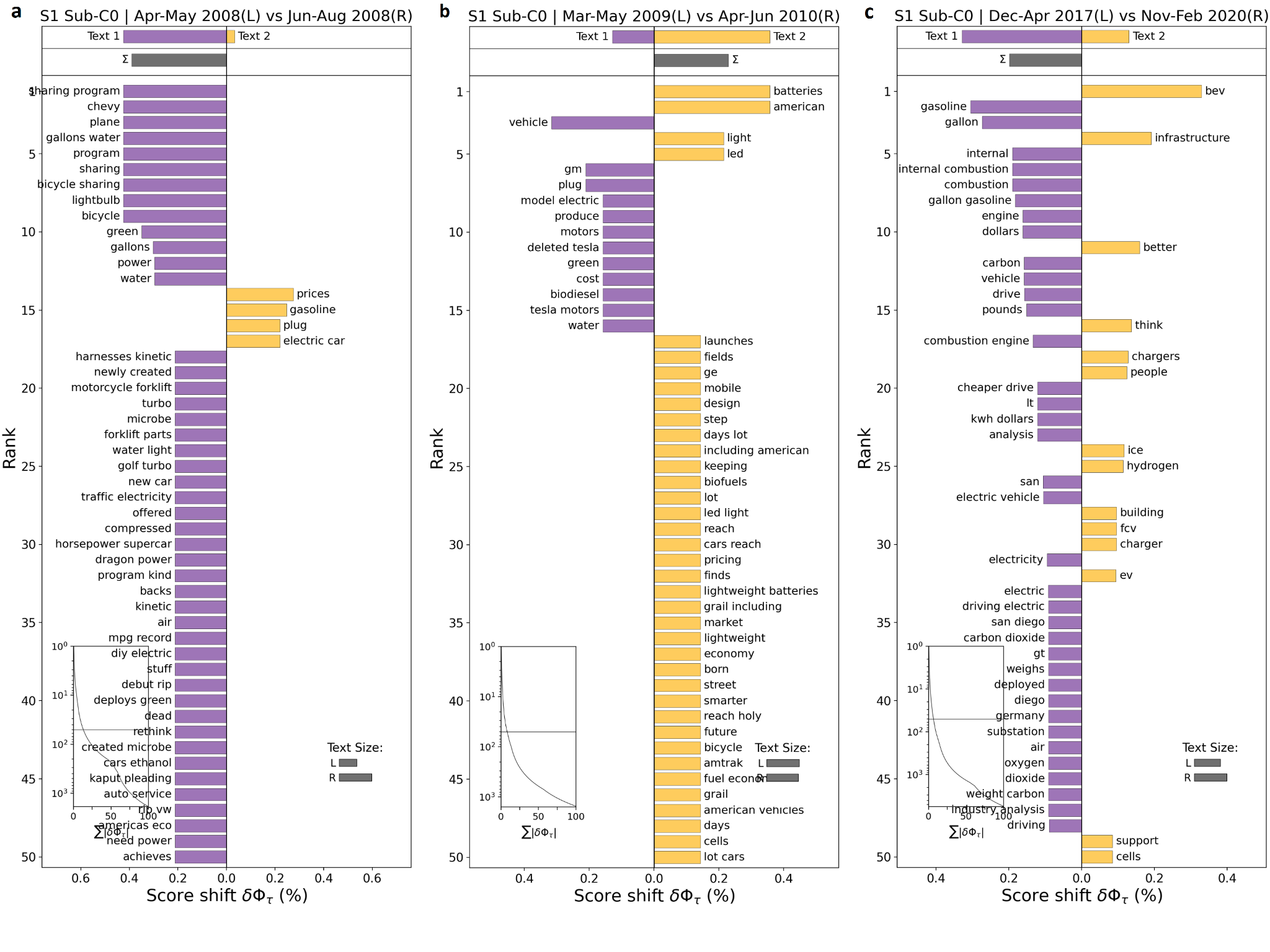


**Extended Data Fig. 5** **| Word shift graphs for *Electric Vehicles/Alternate energy resources* sub-clusters within *Energy* cluster in Sample 1 showing the texts from the time periods with unusually high vs. low user activity.** The two plots (**a, b**) reveal keywords in the discussions around *first biofuel flight take-off* in 2008. The plot on the right (**c**) compares text from two distinct time periods separated by years (2017 to 2020). A closer observation at the text on the left side reveals that in 2017 users were mainly having a debate between “*Internal combustion engine vs. Electric vehicles”*, however, the focus seems to have shifted towards “*fuel cell vehicles (fcv)* and *building better charging infrastructure for batteries* in 2020”*.*


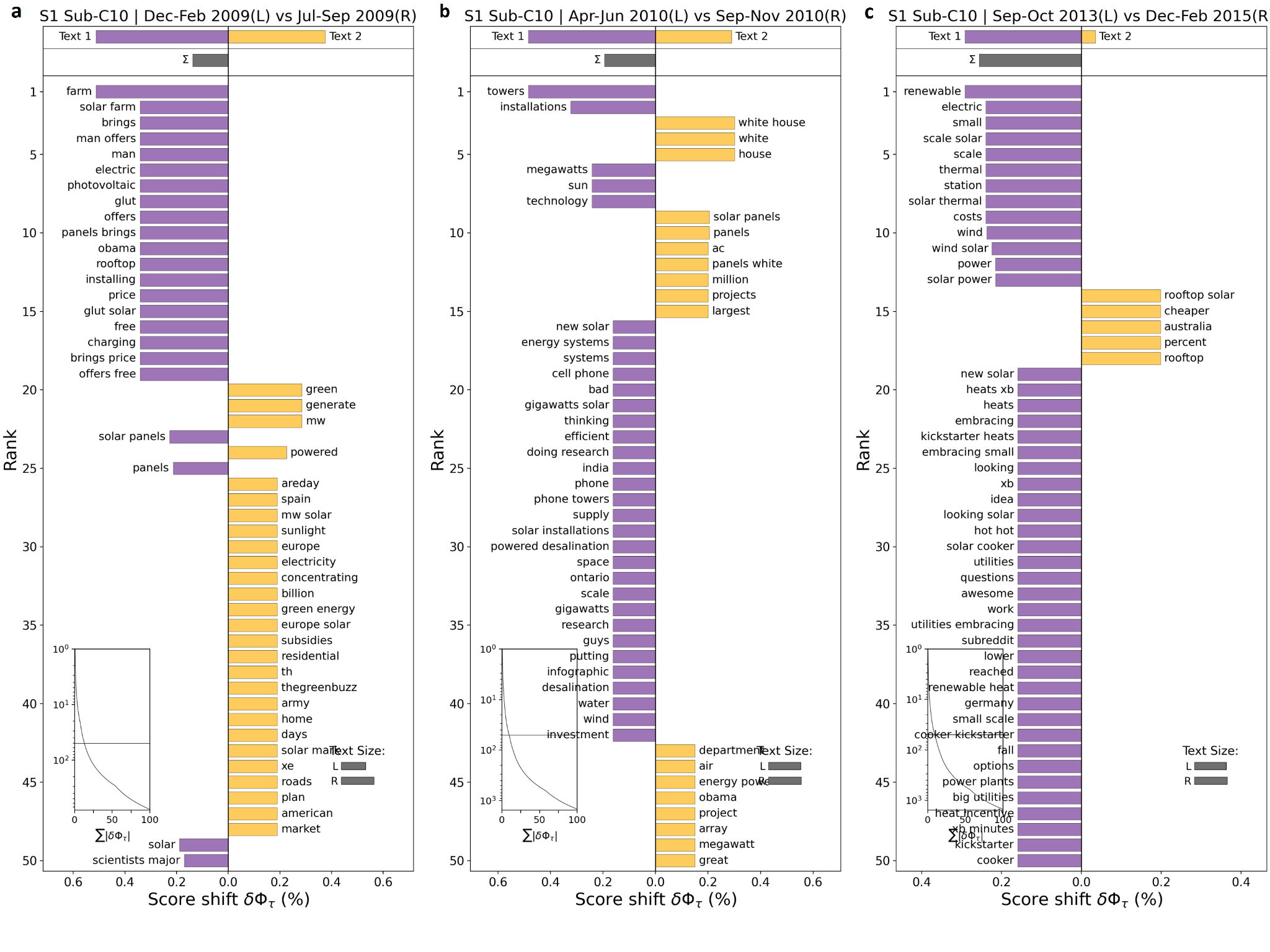


**Extended Data Fig. 6** **| Word shift graphs for *Solar energy* sub-cluster within *Energy* cluster in Sample 1 showing the texts from the time periods with unusually high vs. low user activity.** All three plots show texts from periods in and around some of the key climate related key events when unusually high user activity was observed. Plot on the left (**a**) shows the largest shifts in keywords usage among Reddit users around the announcement of *the EU renewable energy directive 2009/28/EC* in 2009. In the middle plot (**b**)**,** we observe keywords such as “white house”, “solar panels”, “largest” etc. thereby signifying the heightened discussions around the period when the *US approved the world’s largest solar energy project in California* in 2010. Finally, in the right plot (**c**), we observe that although the general discussions continued on plethora of topics, the one theme that stands out is the discussion around the cheap cost of Australian solar rooftop panels after the publication of IEA’s (International Energy Agency) annual report on *Trends in Photovoltaic Applications* in 2015.


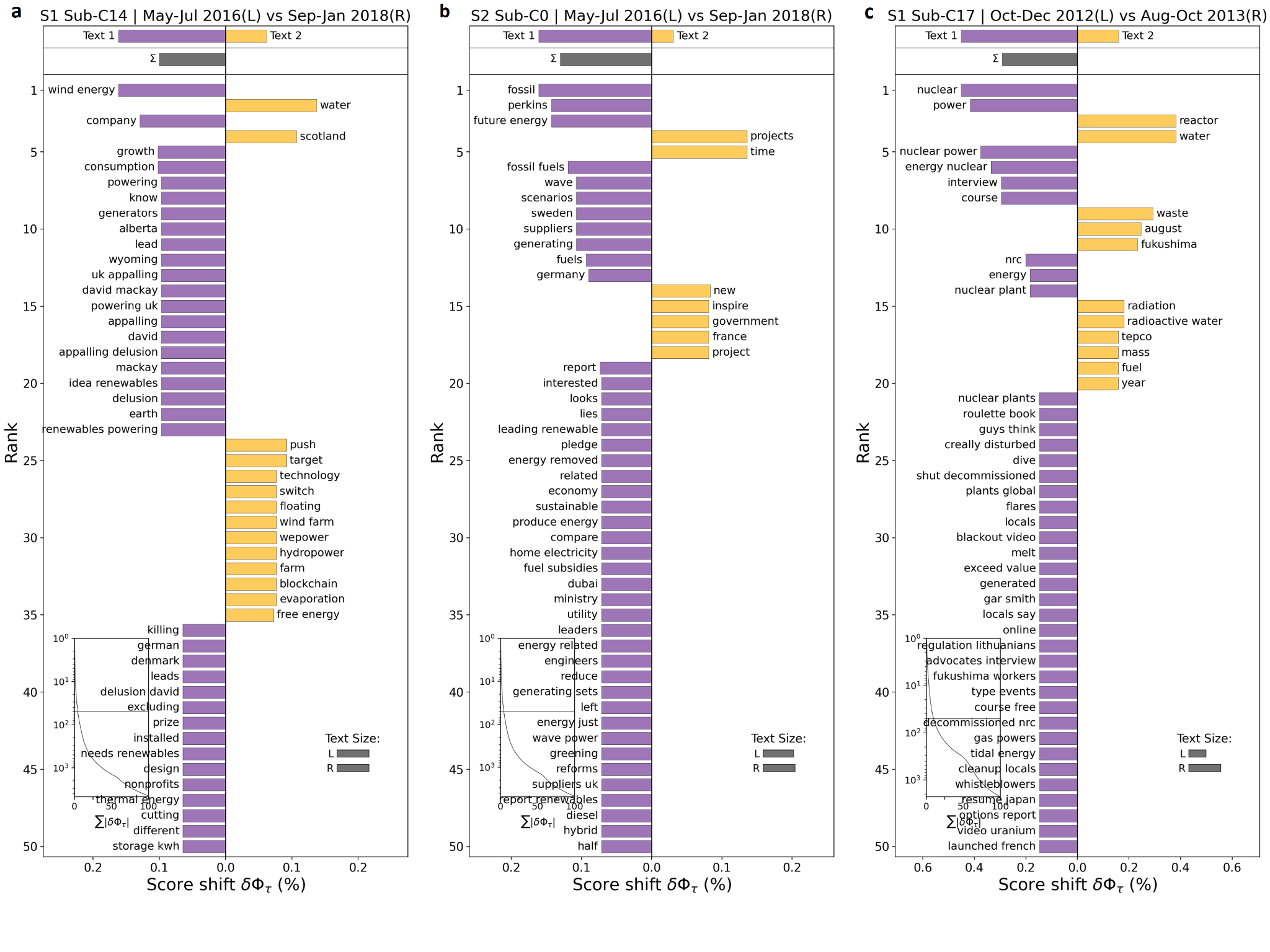


**Extended Data Fig. 7** **| Word shift graphs for *Renewable energy excl. solar* (a, b) and *Nuclear energy (c)* sub-clusters within the *Energy* cluster.** From the two plots on the left (**a, b**), we see that in the *Renewable energy excl. solar* sub-cluster, the discussions mainly focussed on two key events leading to the spike in the discussions in 2017, namely *the public water supplies (Scotland) amendment regulations (****a****)* and *France’s climate plan (****b****).* On the other hand, in the *Nuclear energy* sub-cluster, we observe another spike in discussions in 2013 when *Fukushima’s radioactive water leak* was revealed (**c**).

**
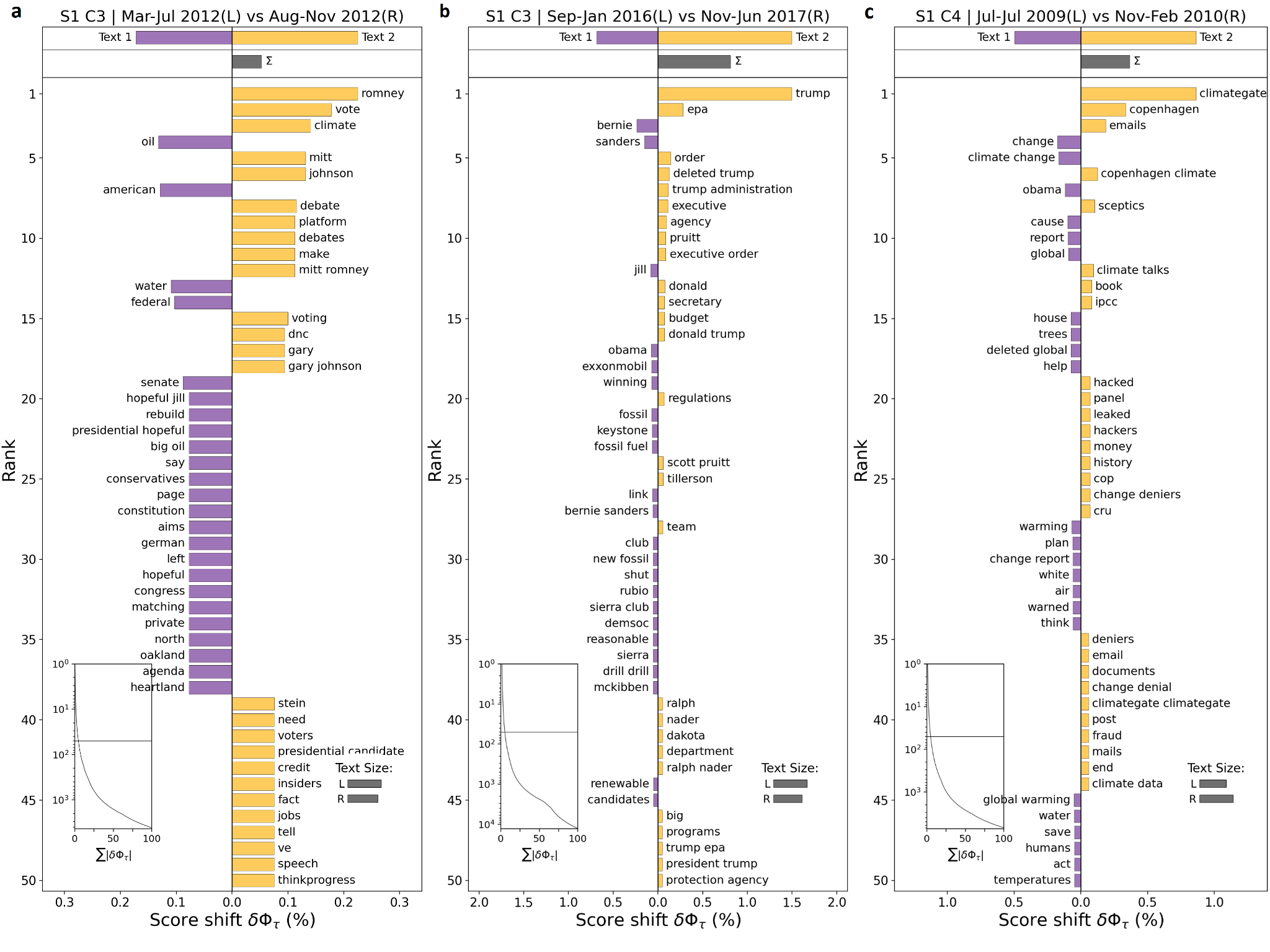
**

**Extended Data Fig. 8 | Word shift graphs for *Administration (a, b)* and *Climate science (c)* clusters showing the texts from the time periods with unusually high vs. low user activity.** The plot on the left shows the keywords around the 2012 US presidential elections, mainly emphasizing the lack of *“climate”* related discussion during presidential debates (**a**). The plot in the middle shows the keywords from user discussions around *2017 People’s climate march,* depicting the protests against the environmental policies of former US President Donal Trump and his administration (**b**). The plot on the right shows keywords within *Climate science* cluster from the period associated with significant rise in discussions around *Copenhagen Climate Change Conference* in 2009 (**c**).


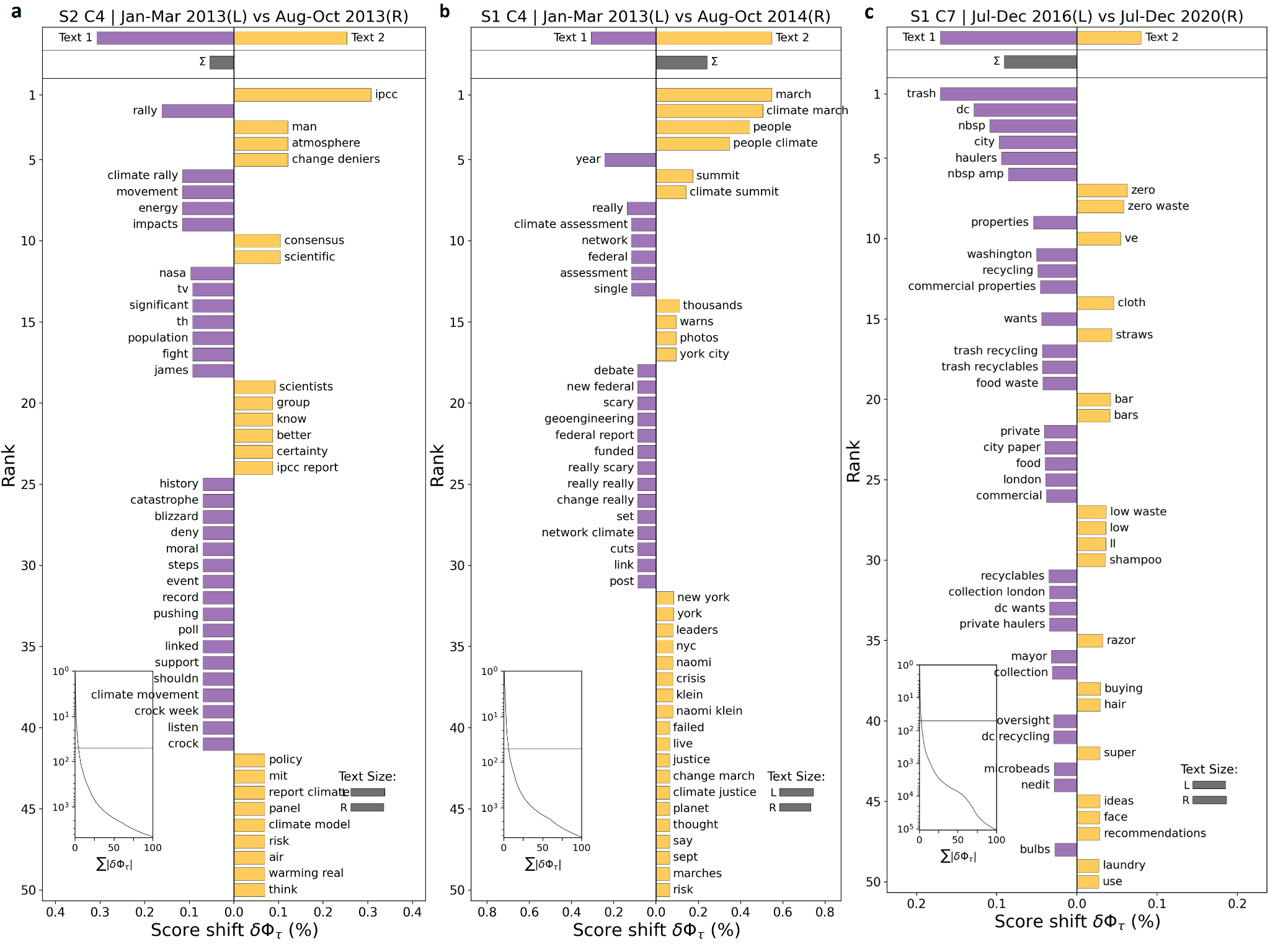


**Extended Data Fig. 9** **| Word shift graphs for *Climate science* (a, b) and *Plastic & waste (c)* clusters.** The plot on the left shows keywords from discussions within the Climate *science* cluster from around the time when IPCC (Intergovernmental Panel on Climate Change) issued its *starkest warning over global warming* in its 2013 report (**a**). Further, the plot in the middle shows keywords from discussions related to 2014 People’s climate march within the same clusters (**b**). Finally, the plot on the left shows keywords within the Plastic *& waste* cluster from the period beyond 2019, after the inclusion of *plastic waste amendments* in the Basel convention (**c**).


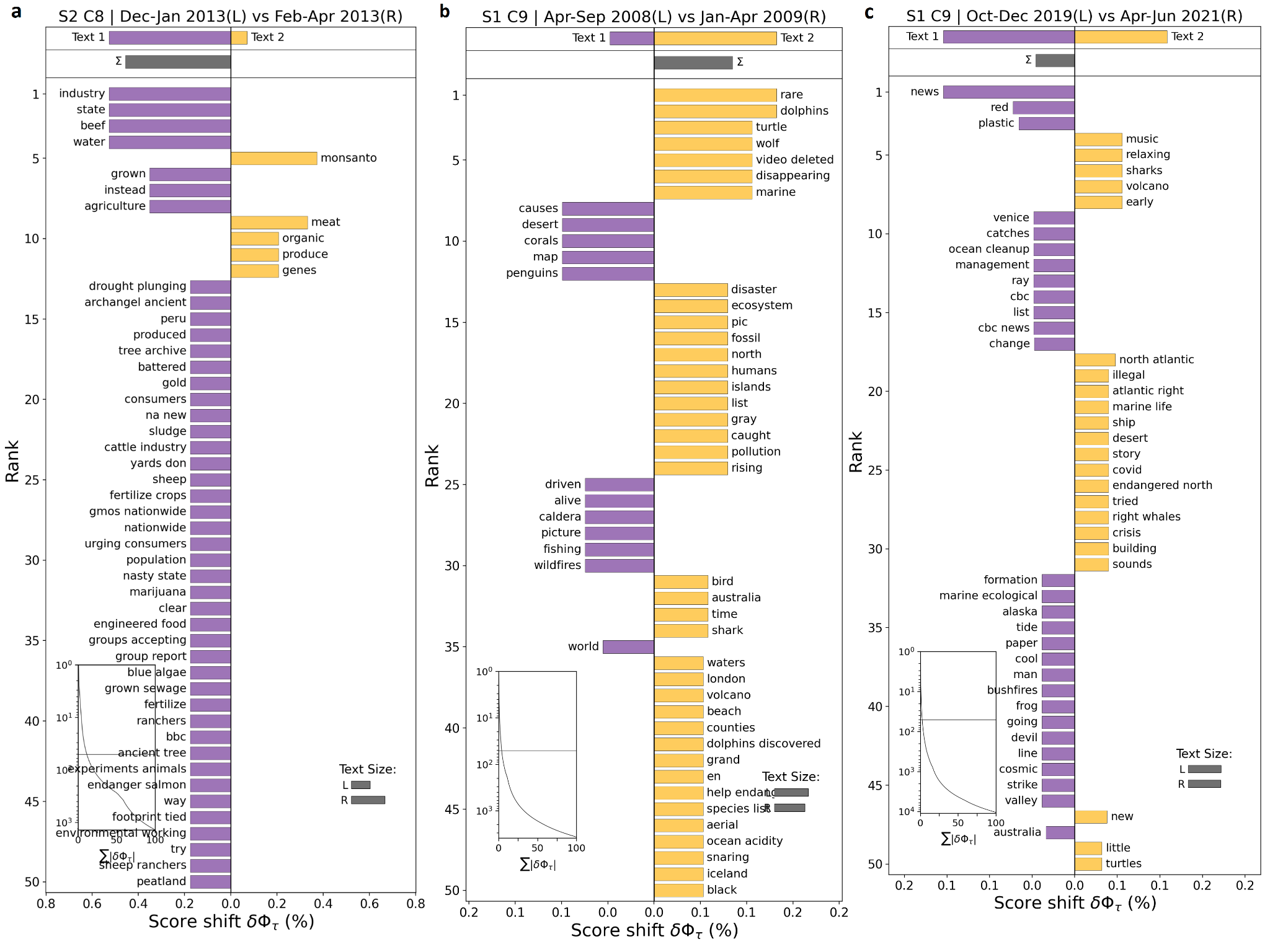


**Extended Data Fig. 10** **| Word shift graphs for *Agriculture* (a) and *Wildlife (b, c)* clusters.** The left plot (**a**) shows the discussions within the *Agriculture* cluster from the period when Section 725, also known as *“the Monsanto Protection Act”* was included in the Consolidated and Further Continuing Appropriations Act, 2013 by the Obama administration. The plots (**b, c**) show two points within *Wildlife* cluster with high user discussions on a variety of topics.


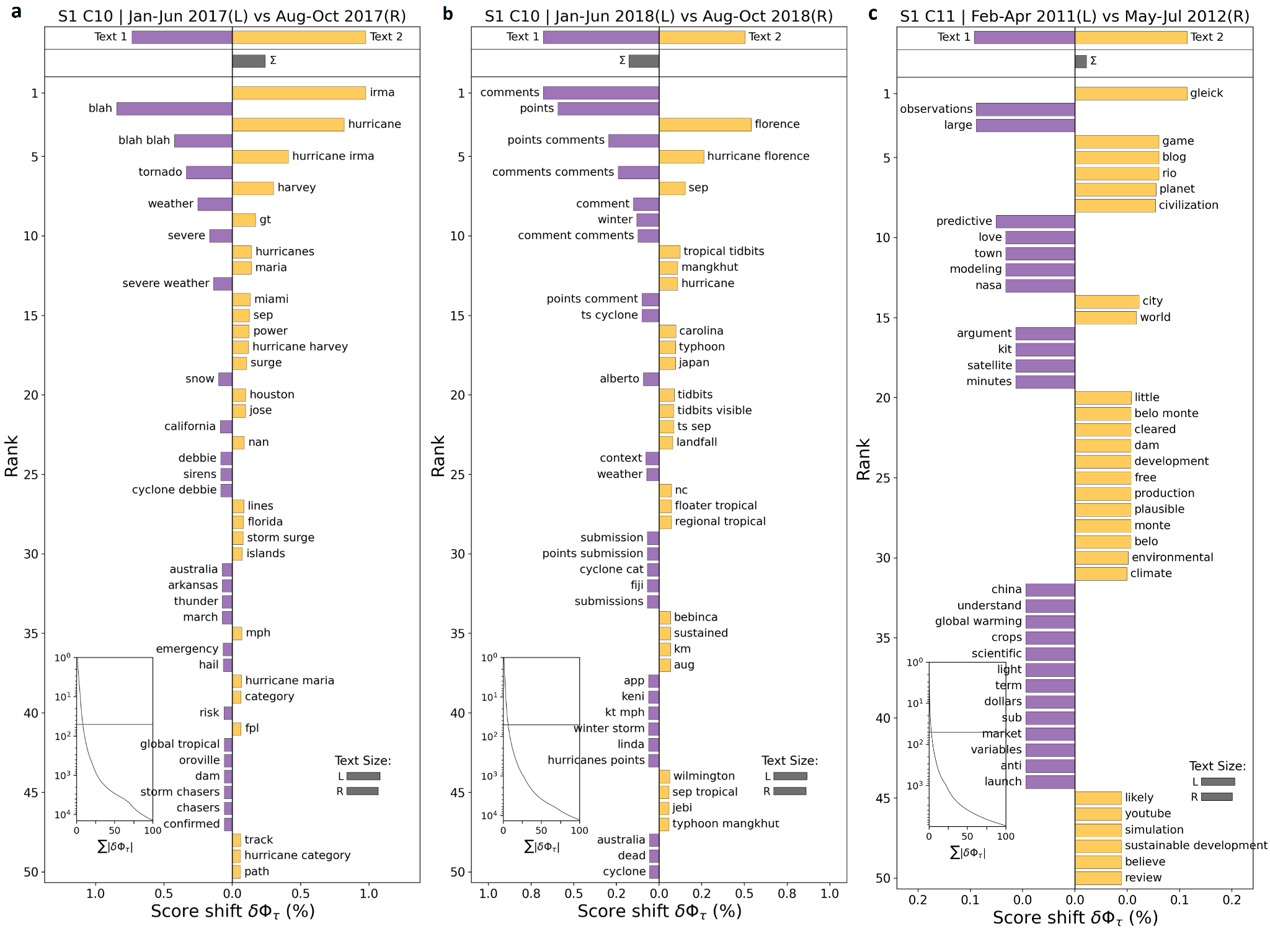


**Extended Data Fig. 11** **| Word shift graphs for *Natural catastrophe* (a, b) and *General posts (c)* clusters.** The two plots on the left (**a, b**), show movement of discussions within the Natural *catastrophe* cluster shifting towards heightened natural catastrophe activities during 2017 and 2018. The plot on the right (**c**), shows the shift in the keywords within the General *posts* cluster from around the time when *Rio+20, UN Conference on Sustainable Development* was held in 2012.


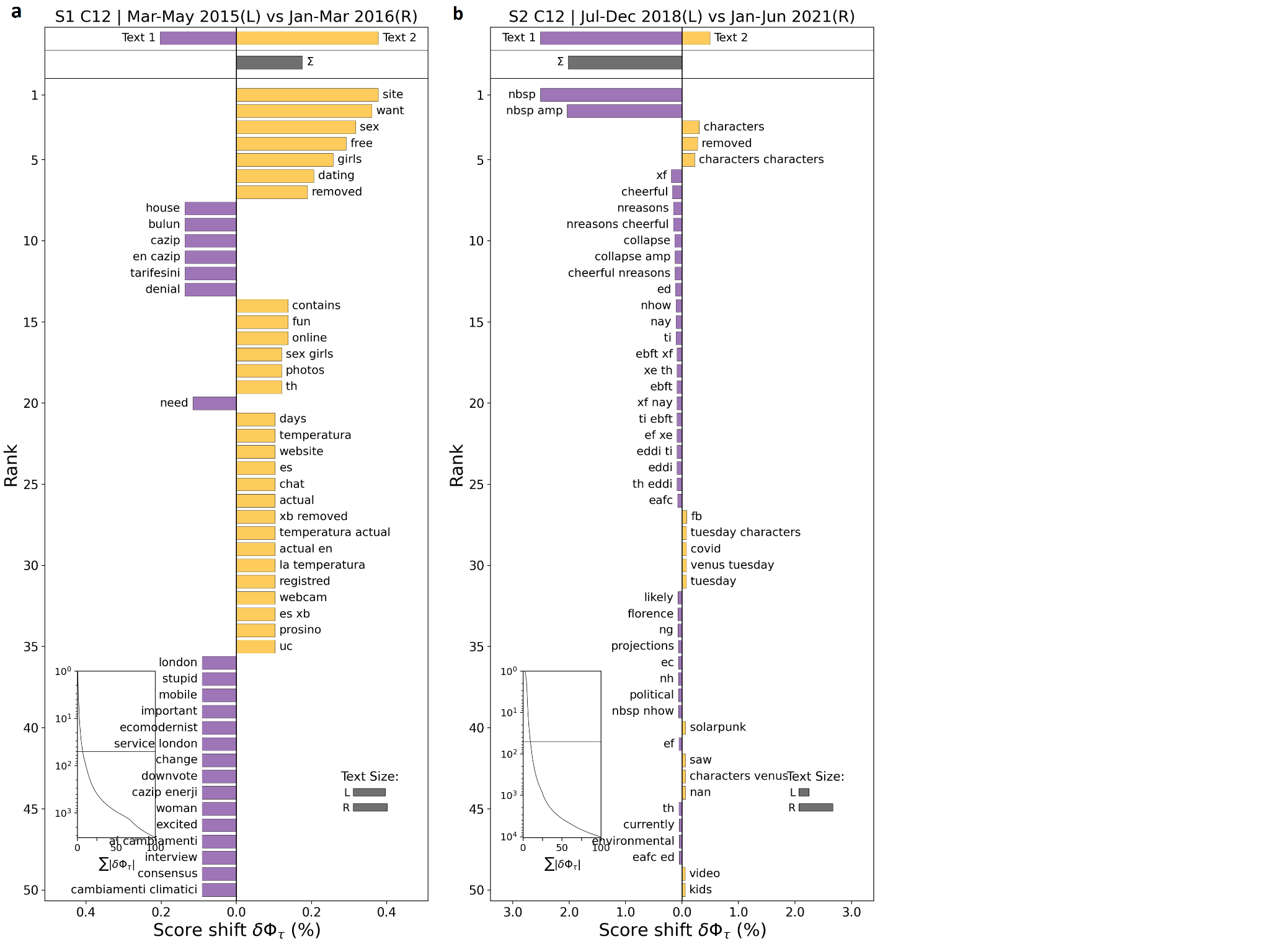


**Extended Data Fig. 12** **| Word shift graphs for the *Unidentifiable* cluster.** The two plots show the shift in keywords within discussions from two time periods when the monthly proportion of discussions peaked in the *Unidentifiable* clusters. However, the keywords do not depict any climate related discussion or event as such.


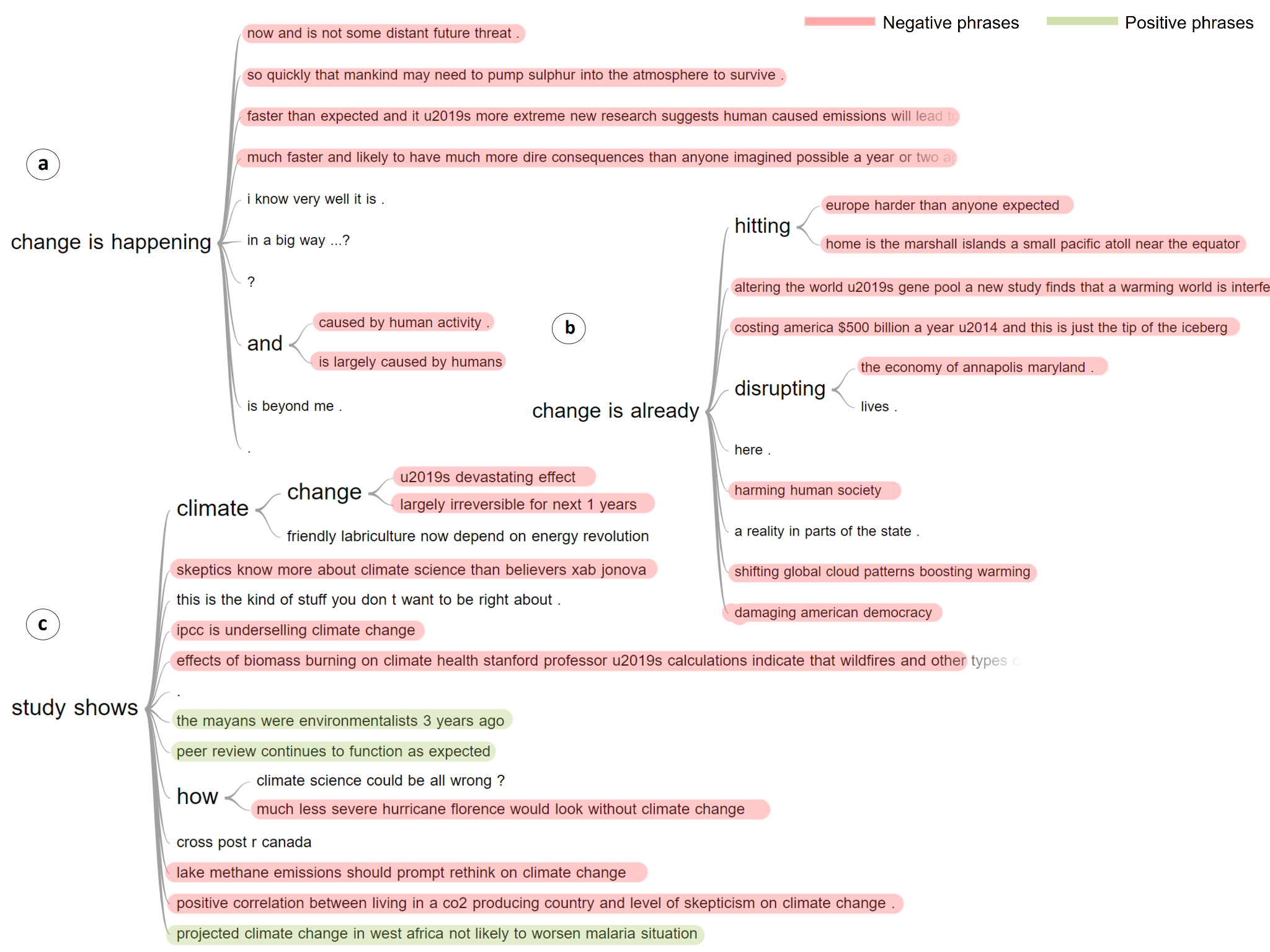


**Extended Data Fig. 13** **| Word trees generated from a set of random phrases taken from the *Climate science* cluster.** Two of the word trees show the discussions around the word *“change”*.in the context of it happening in the present (**a**), and already disrupting the economies around the world (**b**). The third word tree shows the phrases following the keyword *“study shows”* depicting that most of the climate science related studies have negative sentiments associated with it (**c**).


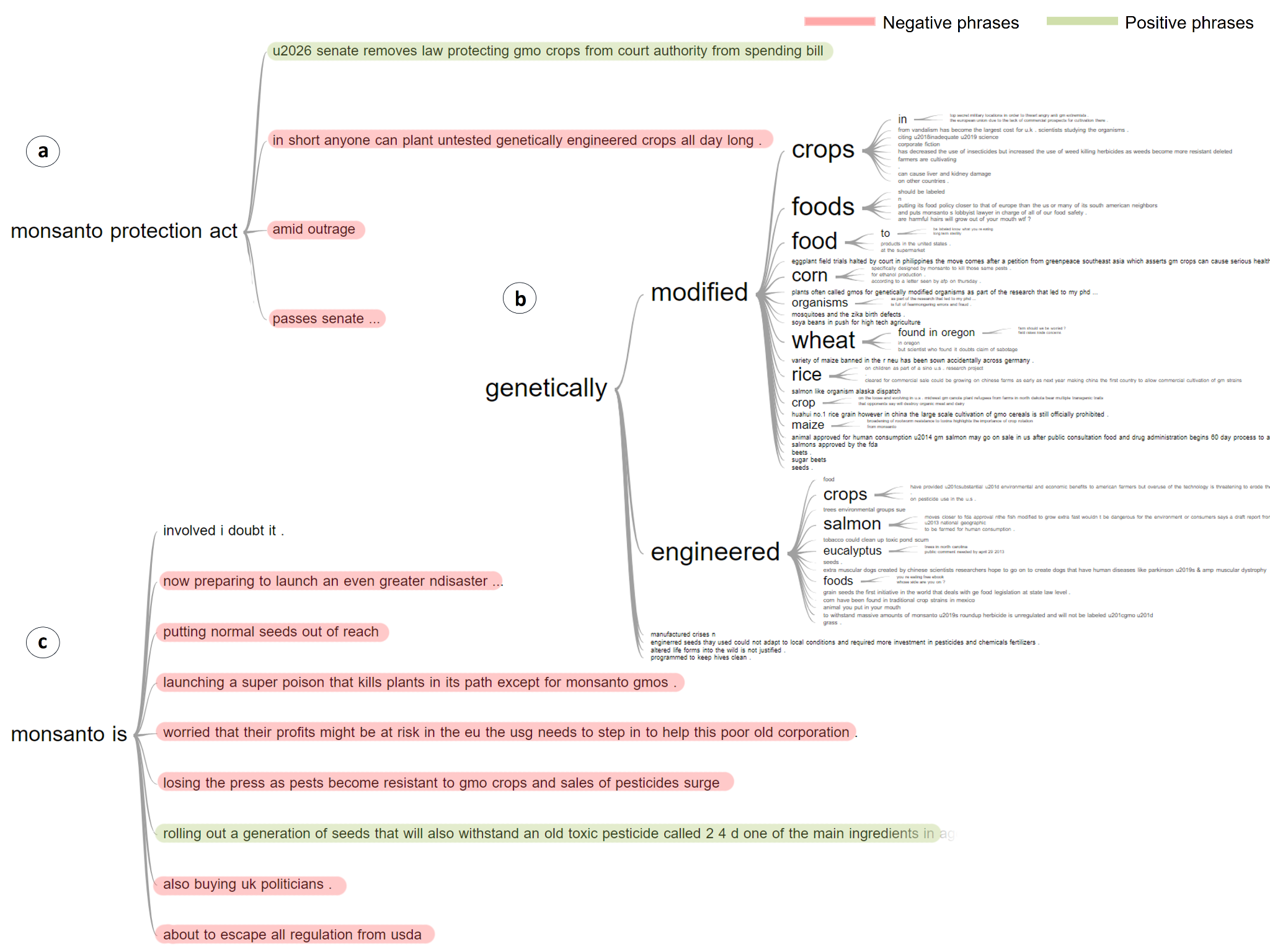


**Extended Data Fig. 14** **| Word trees generated from a set of random phrases taken from the *Agriculture [and administration* for sample 1*]* cluster.** We show the key discussions around the *“Monsanto Protection Act”* (**a**) and the *Monsanto* company (**c**). Also, we note the extensive discussions within the cluster on *genetically modified/engineered crops/foods* (**b**)*.*


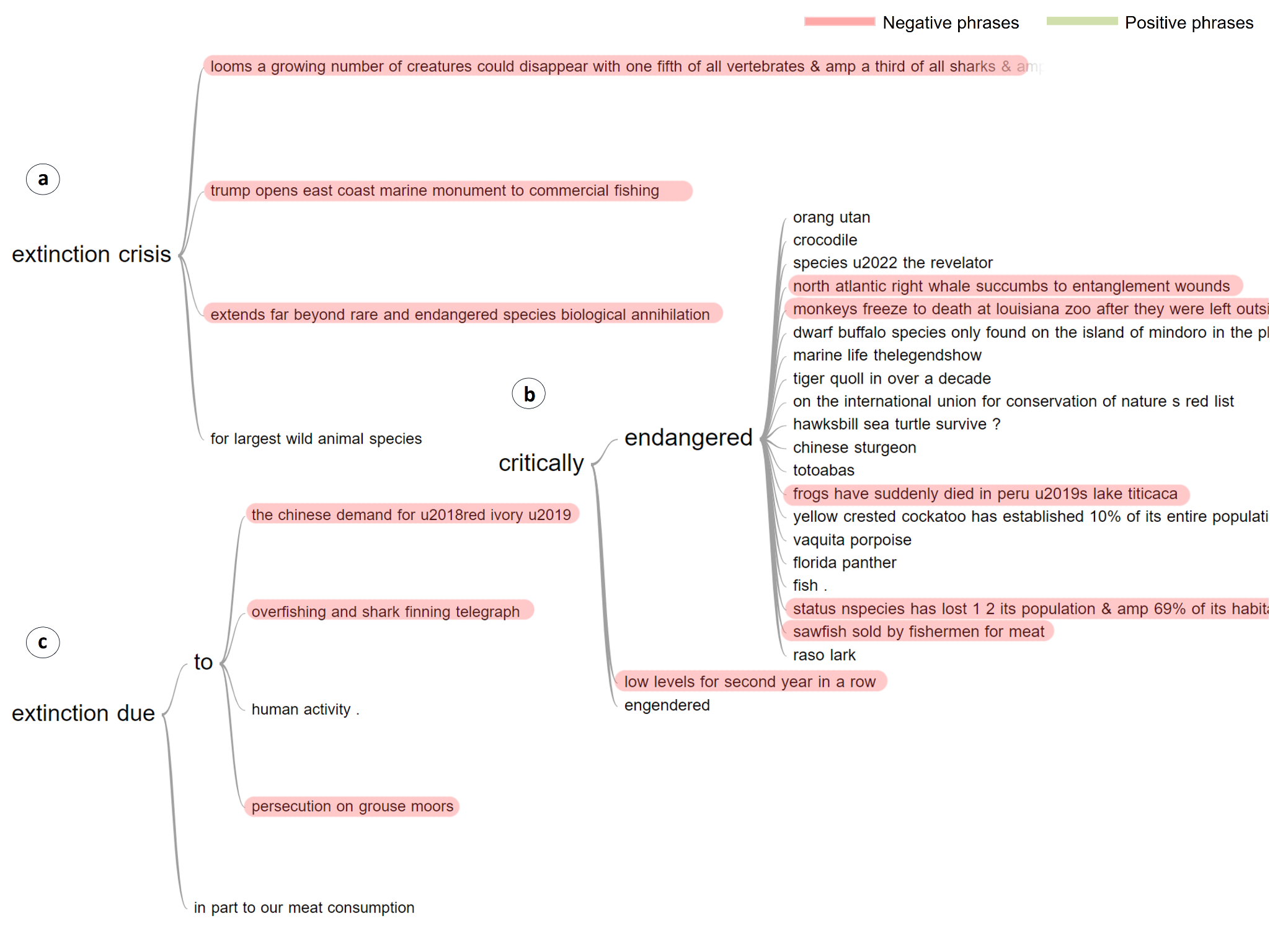


**Extended Data Fig. 15** **| Word trees generated from a set of random phrases taken from the *Wildlife* cluster.** Two of the word trees show the discussions around extinction crisis (**a**) and its causes (**c**). The third word tree shows a range of critically endangered species that were part of the discussions on Reddit (**b**).


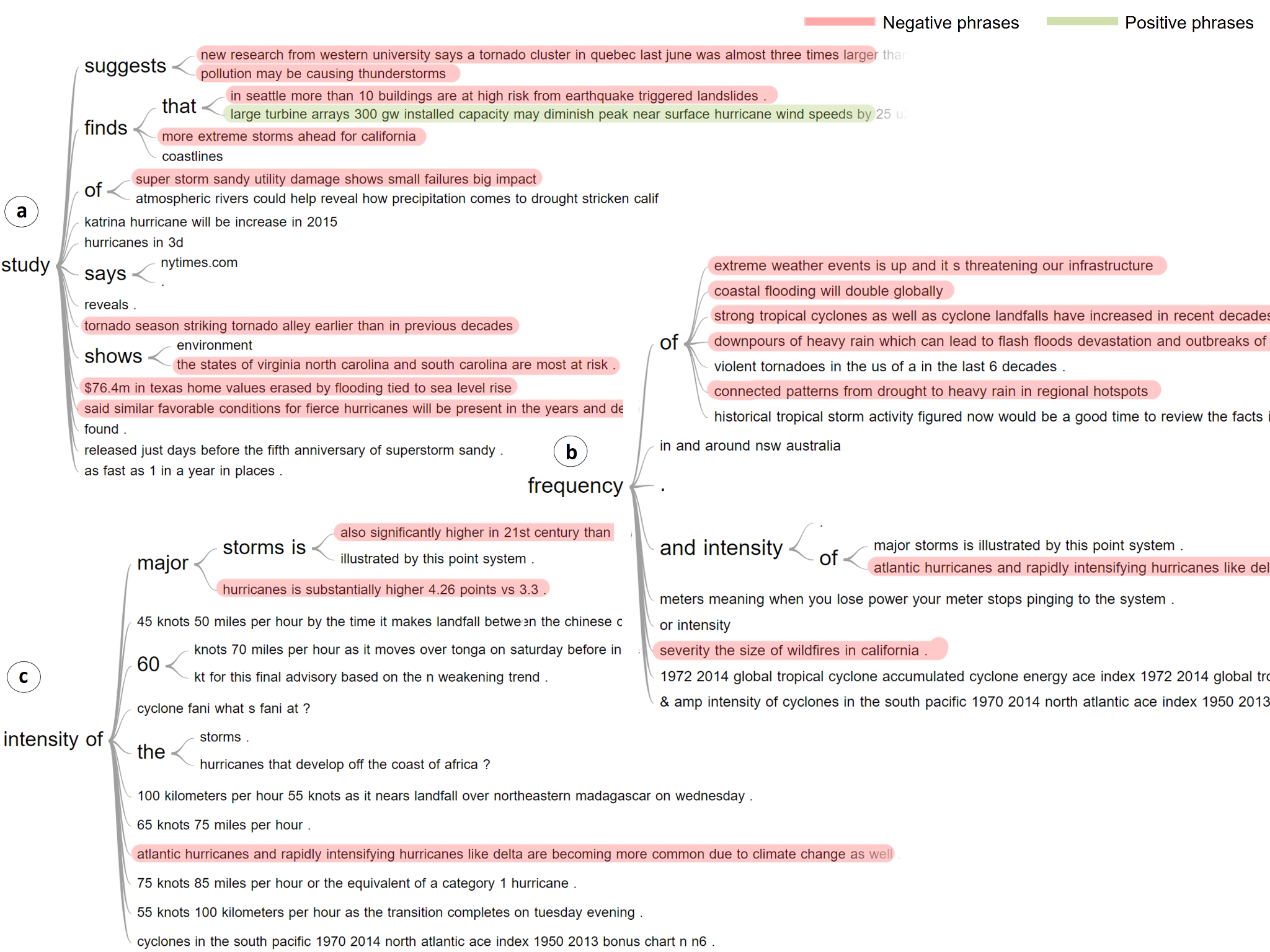


**Extended Data Fig. 16** **| Word trees generated from a set of random phrases taken from the *Natural catastrophe* cluster.** Majority of the studies done related to natural catastrophes and climate change have negative sentiments associated with it (**a**). Further, we observe a lot of discussions around the rising frequency (**b**) and intensity of the natural disasters (**c**).

| **S.No.** | **Subreddit (r/)** | **Date created** | **No. of members in the community (in '000s)** | **Community Description** |
| --- | --- | --- | --- | --- |
| 1 | Environment | Jan 25, 2008 | 1,111 | Current news, information and issues related to the environment. |
| 2 | nature | Jan 25, 2008 | 144 | * Share your fascinating links about nature * Discuss endangered parts of nature * |
| 3 | Green | Jan 25, 2008 | 38 | This subreddit is for issues relating to Green issues, including (but not limited to) Green Politics. |
| 4 | energy | Mar 19, 2008 | 137 | Welcome to energy |
| 5 | collapse | May 02, 2008 | 438 | Discussion regarding the potential collapse of global civilization |
| 6 | climate | May 07, 2008 | 127 | A community for truthful science-based news about climate and related politics and activism |
| 7 | weather | May 21, 2008 | 93 | A community for discussion and posts about weather. Mostly on Earth. |
| 8 | GlobalWarming | May 28, 2008 | 10 | Global warming, global weirding, and what we should do about it. |
| 9 | sustainability | Jul 08, 2008 | 194 | Sustainability is the ability of system to endure. |
| 10 | climateskeptics | Jul 16, 2008 | 34 | Questioning climate related environmentalism. |
| 11 | nuclear | Jul 22, 2008 | 26 | Focus on peaceful use of nuclear energy tech, economics, news, and climate change. |
| 12 | ecology | Nov 13, 2008 | 67 | Ecology is the scientific study of the relations that living organisms have with respect to each other |
| 13 | Sustainable | Jan 05, 2009 | 16 | Sustainable energy, food, water, air, living. |
| 14 | overpopulation | May 15, 2009 | 21 | We are a disease, growing relentlessly, carrying out deforestation and eradication of other species |
| 15 | climatechange | Oct 26, 2009 | 56 | This is a place for the rational discussion of the science of climate change. |
| 16 | ClimateCrisis | Oct 31, 2009 | 1 | Welcome to ClimateCrisis |
| 17 | GreenParty | Jan 21, 2010 | 20 | This is a subreddit for news about Green Parties, Green candidates, and Green politics |
| 18 | transition | Feb 13, 2010 | 3 | The Transition Movement - Communities working towards a sustainable future. |
| 19 | Disasters | May 18, 2010 | 1 | Welcome to /r/Disasters - the hub of everything disaster related. |
| 20 | RenewableEnergy | Jul 23, 2010 | 105 | * Share your fascinating links about renewable energy * Discuss new renewable technologies * |
| 21 | environmental_science | Dec 30, 2010 | 43 | This subreddit is for the *scientific discussion* of topics in the environmental sciences |
| 22 | GreenTechnology | May 23, 2011 | 0 | Welcome to GreenTechnology |
| 23 | TropicalWeather | Sep 07, 2011 | 111 | This is a subreddit designed for all sorts of tropical cyclone weather discussion. |
| 24 | ZeroWaste | Feb 20, 2013 | 839 | We are responsible citizens who try to minimize our overall environmental impact. |
| 25 | SaveTheEnvironment | Feb 21, 2013 | 0 | Post environmentally friendly ideas here! Help the world, one post at a time. |
| 26 | enviroaction | Feb 23, 2013 | 11 | A subreddit dedicated to Environmental *Action* - petitions, fundraisers, events and other actions |
| 27 | carboncapture | Feb 25, 2013 | 1 | Discussion of CO2 capture, transport and storage. |
| 28 | EarthDisaster | Feb 28, 2013 | 1 | The purpose of this community is for analysis, notification and discussion of disasters |
| 29 | carbontax | Jul 11, 2014 | 2 | If you ask economists how to stop global warming, they'd probably recommend taxing carbon. |
| 30 | GlobalClimateChange | Jul 20, 2014 | 4 | A place to share information, thoughts and ideas, news, and credible studies and research |
| 31 | climatejustice | Sep 22, 2014 | 2 | News and discussion from the climate justice movement. |
| 32 | solarpunk | Nov 02, 2014 | 91 | Solarpunk is a genre and aesthetic that envisions collective futures that are vibrant with life |
| 33 | climate_science | Feb 20, 2015 | 20 | This community is a repository for peer-reviewed climate change analysis and expert commentary |
| 34 | CitizensClimateLobby | Jun 23, 2016 | 14 | We exist to empower Redditors to have breakthroughs for meaningful climate legislation. |
| 35 | climate_discussion | Oct 05, 2016 | 2 | /r/Climate_Discussion is a subreddit having an ongoing discussion about climate change |
| 36 | EcoInternet | Jan 04, 2017 | 3 | Climate Change and Environment News |
| 37 | ClimateMobilization | Apr 05, 2017 | 0 | Movement for Climate Mobilization |
| 38 | ClimateChangeSurprise | Nov 22, 2017 | 0 | This is a subreddit to chronicle and discuss unpredictable consequences of global climate decay |
| 39 | CarbonFootprint | Jul 10, 2018 | 0 | A community dedicated to providing everyday people with easy steps to reduce their carbon footprint |
| 40 | EcoNewsNetwork | Jul 16, 2018 | 7 | Information, appreciation, discussion, links to articles, websites, images, videos, from reputable sites |
| 41 | ClimateActionPlan | Aug 19, 2018 | 81 | A news based subreddit highlighting active measures to combat/mitigate and or adapt to climate change. |
| 42 | saveplanetearth | Sep 18, 2018 | 2 | A subreddit for promoting the conservation of our planet. |
| 43 | sustainability2 | Oct 19, 2018 | 0 | Sustainability is key |
| 44 | ClimateOffensive | Oct 29, 2018 | 62 | We're here to brainstorm, organize, and act. Use this space to find resources, connect with others |
| 45 | ExtinctionRebellion | Oct 29, 2018 | 23 | We are in the sixth mass extinction event and we will face catastrophe if we do not act swiftly. |
| 46 | EarthStrike | Nov 11, 2018 | 22 | Environmental movement focused on organising a GLOBAL GENERAL STRIKE TO SAVE THE PLANET! |
| 47 | SunriseMovement | Dec 06, 2018 | 3 | Sunrise is a movement to stop climate change and create millions of good jobs in the process. |
| 48 | FridaysForFuture | Jan 25, 2019 | 4 | A sub dedicated to the international movement of students to demand action to prevent climate change. |
| 49 | EarthApproach | Feb 11, 2019 | 0 | Our goal is to create practical and sustainable approaches to human existence |
| 50 | ClimateCrisisCanada | May 03, 2019 | 3 | A place to share posts and comments on the global climate crisis from the perspective of Canadians |
| 51 | ClimateRealityProject | May 03, 2019 | 1 | Our mission is to catalyze a global solution to the climate crisis by making urgent action a necessity |
| 52 | SaveEarth | Jun 03, 2019 | 0 | SaveEarth is a subreddit which aims towards taking steps to prevent our planet from drying out. |
| 53 | EnvironmentProtectors | Dec 14, 2019 | 1 | A largely unmoderated Subreddit for everything to do with saving the environment, planet |
| 54 | NaturalCatastrophe | Mar 11, 2021 | 0 | The Planet Today News from the world of Natural catastrophe. |
| 55 | IndianPrakrti | May 29, 2021 | 1 | Anything related to Indian environment, ecology, wildlife, green jobs & news and activism |

**Extended Table. 1** **| Table providing details on the 55 climate related subreddits selected from Reddit for data collection.**
